# Glucose-Raising Polymorphisms in the Human Clock Gene Cryptochrome 2 (*CRY2*) Affect Hepatic Lipid Content

**DOI:** 10.1371/journal.pone.0145563

**Published:** 2016-01-04

**Authors:** Fausto Machicao, Andreas Peter, Jürgen Machann, Ingmar Königsrainer, Anja Böhm, Stefan Zoltan Lutz, Martin Heni, Andreas Fritsche, Fritz Schick, Alfred Königsrainer, Norbert Stefan, Hans-Ulrich Häring, Harald Staiger

**Affiliations:** 1 Institute for Diabetes Research and Metabolic Diseases of the Helmholtz Centre Munich at the University of Tübingen, Tübingen, Germany; 2 German Center for Diabetes Research (DZD), Tübingen, Germany; 3 Department of Internal Medicine, Division of Endocrinology, Diabetology, Angiology, Nephrology and Clinical Chemistry, University Hospital Tübingen, Tübingen, Germany; 4 Department of Diagnostic and Interventional Radiology, Section on Experimental Radiology, University Hospital Tübingen, Tübingen, Germany; 5 Department of General, Visceral, and Transplant Surgery, University Hospital Tübingen, Tübingen, Germany; 6 Department of Internal Medicine, Division of Nutritional and Preventive Medicine, University Hospital Tübingen, Tübingen, Germany; GDC, GERMANY

## Abstract

Circadian rhythms govern vital functions. Their disruption provokes metabolic imbalance favouring obesity and type-2 diabetes. The aim of the study was to assess the role of clock genes in human prediabetes. To this end, genotype-phenotype associations of 121 common single nucleotide polymorphisms (SNPs) tagging *ARNTL*, *ARNTL2*, *CLOCK*, *CRY1*, *CRY2*, *PER1*, *PER2*, *PER3*, and *TIMELESS* were assessed in a study population of 1,715 non-diabetic individuals metabolically phenotyped by 5-point oral glucose tolerance tests. In subgroups, hyperinsulinaemic-euglycaemic clamps, intravenous glucose tolerance tests, and magnetic resonance imaging/spectroscopy were performed. None of the tested SNPs was associated with body fat content, insulin sensitivity, or insulin secretion. Four *CRY2* SNPs were associated with fasting glycaemia, as reported earlier. Importantly, carriers of these SNPs’ minor alleles revealed elevated fasting glycaemia and, concomitantly, reduced liver fat content. In human liver tissue samples, *CRY2* mRNA expression was directly associated with hepatic triglyceride content. Our data may point to *CRY2* as a novel switch in hepatic fuel metabolism promoting triglyceride storage and, concomitantly, limiting glucose production. The anti-steatotic effects of the glucose-raising *CRY2* alleles may explain why these alleles do not increase type-2 diabetes risk.

## Introduction

Circadian rhythms control behavioural aspects, such as sleeping/waking, eating, and socializing. Modern lifestyle, characterized by flexible working hours, long-distance flights, and 24-h service offerings, increasingly disrupts this rhythmicity. Disruption of circadian rhythms does not only affect neurotransmitter release in the brain, but also impairs cellular metabolism in peripheral organs promoting metabolic imbalance of the whole body [1]. Thus, night shift workers experience not only mental and social changes but also increments in abdominal fat mass and plasma glucose concentrations and emergence of atherogenic plasma lipid profiles that promote the development of the metabolic syndrome, type-2 diabetes, and coronary heart disease [2–4].

The endogenous clock is hierarchically organized with a central clock located in the suprachiasmatic nucleus that indirectly senses light and synchronizes the peripheral clocks via neuronal and humoral signals [5,6]. Molecularly, the central and peripheral clocks are similarly composed of oscillating concentrations of transcriptional regulators [7,8]: the core clock is formed by the transcription factor heterodimer CLOCK (circadian locomotor output cycles kaput)—BMAL1 (brain and muscle aryl hydrocarbon receptor nuclear translocator-like [ARNTL] protein 1). Among the hundreds of target genes of CLOCK-BMAL1 are the cryptochrome (*CRY*) genes *CRY1* and *CRY2* and the period (*PER*) genes *PER1*, *PER2*, and *PER3*. Following expression, CRY-PER heterodimers assemble and act as corepressors by binding to and removing the CLOCK-BMAL1 complex from promoters and enhancers, thereby switching off CLOCK-BMAL1-induced gene transcription. In consequence, *CRY* and *PER* expression drops again and CLOCK-BMAL1 activity is restored. This oscillating core machinery is complemented by a complex network of additional levels of regulation. For instance, AMP-activated protein kinase senses low energy load in the cell and, in response, phosphorylates and activates casein kinase Iε [9,10]. Active casein kinase Iε phosphorylates CRY-PER, and this leads to CRY-PER degradation, thereby relieving the inhibition of CLOCK-BMAL1 [11]. Moreover, novel clock genes were identified via homology screens, such as *ARNTL2* and *TIMELESS* (timeless circadian clock) [12–15], but their exact biological roles are hitherto not well understood.

Few candidate gene studies reported associations of single nucleotide polymorphisms (SNPs) in *CLOCK*, *ARNTL* (encoding BMAL1), and *PER2* with risk factors for the metabolic syndrome and type-2 diabetes, such as abdominal obesity, hypertension, fatty liver, and an atherogenic lipid profile [16–22]. Moreover, recent genome-wide association (GWA) studies revealed fasting glucose-raising variants in *CRY2* which, surprisingly, were not robustly associated with type-2 diabetes [23,24]. Since the role of clock genes in the development of human type-2 diabetes is still unclear, we assessed the impact of common genetic variation in these genes on prediabetic traits in a human study population at increased risk for type-2 diabetes. We genotyped 121 common SNPs tagging *ARNTL*, *ARNTL2*, *CLOCK*, *CRY1*, *CRY2*, *PER1*, *PER2*, *PER3*, and *TIMELESS* and assessed their impact on glycaemia, body fat content, body fat distribution, insulin sensitivity, and insulin release.

## Materials and Methods

### Ethics Statement

All procedures followed were in accordance with the ethical standards of the responsible committee on human experimentation (Ethics Committee of the Eberhard Karls University Tübingen) and with the Helsinki Declaration of 1975, as revised in 2000. The study protocol was approved by the Ethics Committee, and informed written consent was obtained from all participants for being included in the study.

### Study Participants

The overall study population consisted of 1,715 German individuals recruited from the ongoing Tübingen Family (TÜF) study for type-2 diabetes which currently comprises more than 2,500 non-related individuals at increased risk for type-2 diabetes, i.e., non-diabetic subjects with family history of type-2 diabetes, BMI ≥ 27 kg/m^2^, impaired fasting glycaemia, and/or previous gestational diabetes [25]. All TÜF participants underwent assessment of medical history, smoking status, and alcohol consumption habits, physical examination, routine blood tests, and oral glucose tolerance tests (OGTTs). The overall study population consisted of individuals with complete OGTT and genotype data sets and documented absence of medication known to influence glucose tolerance, insulin sensitivity, or insulin secretion. From 1,682 and 1,632 subjects of the overall study population, respectively, bioelectric impedance measurements and C-peptide data were available. Subgroups of 518, 375, 357, and 314 subjects agreed to undergo hyperinsulinaemic-euglycaemic clamps (HECs), magnetic resonance spectroscopy (MRS) of the liver, whole-body magnetic resonance imaging (MRI), and/or intravenous glucose tolerance tests (IVGTTs), respectively. The clinical characteristics of the overall study population and the subgroups are presented in Table 1.

**Table 1:** Clinical data of the overall study population and the subgroups.

Parameter	Overall population (N = 1,715)	Bioimpedance (N = 1,682)	C-Peptide data (N = 1,632)	HEC (N = 518)	MRS (N = 375)	MRI (N = 357)	IVGTT (N = 314)
N (women/men)	1,122/593	1,105/577	1,072/560	274/244	238/137	222/135	180/134
Age (y)	39 ± 12	39 ± 12	39 ± 12	40 ± 12	45 ± 12	45 ± 12	45 ± 11
BMI (kg/m²)	30.0 ± 9.1	29.9 ± 8.9	30.2 ± 9.2	27.3 ± 5.7	30.1 ± 5.1	30.0 ± 5.3	29.4 ± 5.7
NGT/IFG/IGT/IFG+IGT	1,211/194/162/148	1,193/189/166/134	1,148/187/153/144	397/40/48/33	236/50/48/41	225/43/49/40	210/32/43/29
Glucose, fasting (mmol/L)	5.14 ± 0.55	5.13 ± 0.55	5.14 ± 0.56	5.02 ± 0.53	5.26 ± 0.50	5.23 ± 0.50	5.18 ± 0.49
2-h Glucose (mmol/L)	6.34 ± 1.65	6.34 ± 1.64	6.36 ± 1.64	6.17 ± 1.70	6.93 ± 1.54	6.92 ± 1.58	6.77 ± 1.66
ISI, OGTT (x 10^19^ L^2^ x mol^-2^)	15.5 ± 10.7	15.7 ± 10.7	15.5 ± 10.8	18.2 ± 11.2	12.3 ± 6.7	12.6 ± 6.9	14.0 ± 7.9
Body fat content (%)	-	32.4 ± 12.0	32.4 ± 12.0	28.2 ± 9.7	33.7 ± 9.2	33.0 ± 8.9	31.8 ± 8.9
AUC_Ins0-30_/AUC_Glc0-30_ (x 10^−9^)	-	-	43.8 ± 32.4	37.2 ± 24.8	43.1 ± 27.1	42.3 ± 27.2	40.6 ± 24.9
AUC_C-Pep0-120_/AUC_Glc0-120_ (x 10^−9^)	-	-	321 ± 104	311 ± 97	306 ± 87	306 ± 90	307 ± 92
ISI, HEC (x 10^6^ L x kg^-1^ x min^-1^)	-	-	-	0.084 ± 0.052	-	-	0.069 ± 0.041
IHL (% signal)	-	-	-	-	5.99 ± 6.49	5.96 ± 6.46	-
TAT (% body weight)	-	-	-	-	-	30.5 ± 9.1	-
VAT (% body weight)	-	-	-	-	-	3.33 ± 1.70	-
AIR (pmol/L)	-	-	-	-	-	-	933 ± 629

Data are given as counts or means ±SD. AIR—acute insulin response; AUC—area under the curve; BMI—body mass index; C-Pep—C-peptide; Glc—glucose; HEC—hyperinsulinaemic-euglycaemic clamp; IFG—impaired fasting glycaemia; IGT—impaired glucose tolerance; IHL—intrahepatic lipids; Ins—insulin; ISI—insulin sensitivity index; IVGTT—intravenous glucose tolerance test; MRI—magnetic resonance imaging; MRS—magnetic resonance spectroscopy; NGT—normal glucose tolerance; OGTT—oral glucose tolerance test; TAT—total adipose tissue; VAT—visceral adipose tissue

### Clinical Tests

A standardised 75-g OGTT was performed following a 10-h overnight fast. For the determination of plasma glucose, insulin, and C-peptide levels, venous blood samples were drawn at baseline and at time-points 30, 60, 90, and 120 min of the OGTT [25]. In those subjects who agreed to undergo both the IVGTT and the HEC, the IVGTT was performed after a 10-h overnight fast and prior to the HEC according to the Botnia protocol [26]. After collection of baseline samples (-10 and -5 min), a glucose dose of 0.3 g/kg body weight was given at time-point 0. Blood samples for the measurement of plasma glucose and insulin were obtained at 2, 4, 6, 8, 10, 20, 30, 40, 50, and 60 min of the IVGTT. The HEC was performed starting at 60 min after the IVGTT glucose bolus. To this end, subjects received a primed infusion of short-acting human insulin (40 mU/m^2^/min) for 120 min. A variable infusion of 20% glucose was started to clamp the plasma glucose concentration at fasting levels. Blood samples for the measurement of plasma glucose were obtained at 5-min intervals. Plasma insulin levels were measured at baseline (prior to the IVGTT glucose bolus) and at the steady state (the last 30 min) of the clamp. In subjects who agreed to undergo the HEC, but not the IVGTT, the HEC was started immediately after the 10-h overnight fast.

### Measurements of Body Fat Content and Body Fat Distribution

BMI was calculated as weight divided by squared height (in kg/m^2^), the percentage of body fat was measured by bioimpedance (BIA-101, RJL systems, Detroit, MI, USA). Whole-body MRI was performed to determine total adipose tissue (TAT) and visceral adipose tissue (VAT) contents [27]. The intrahepatic lipid (IHL) content was quantified by localized stimulated echo acquisition mode ^1^H-MRS [28].

### Laboratory Measurements

Plasma glucose (in mmol/L) was measured with a bedside glucose analyser (glucose oxidase method, Yellow Springs Instruments, Yellow Springs, OH, USA). Serum insulin and C-peptide (in pmol/L, both) were determined by commercial chemiluminescence assays for ADVIA Centaur (Siemens Medical Solutions, Fernwald, Germany).

### Calculations

The OGTT-derived insulin sensitivity index (ISI OGTT) was calculated as 10,000/[c(Glc_0_) x c(Ins_0_) x c(Glc_mean_) x c(Ins_mean_)]^½^ (with c = concentration, Glc = glucose, and Ins = insulin) [29]. The HEC-derived ISI (ISI HEC) was calculated as glucose infusion rate necessary to maintain euglycaemia during the last 20 min (steady state) of the clamp (in μmol/kg/min) divided by the steady-state insulin concentration (in pmol/L). Insulin secretion was estimated by three indices: OGTT-derived insulin secretion was calculated as area under the curve (AUC)_Ins0-30_/AUC_Glc0-30_ and AUC_C-Pep0-120_/AUC_Glc0-120_ (with C-Pep = C-peptide). AUC_Ins0-30_/AUC_Glc0-30_ was calculated as [c(Ins_0_) + c(Ins_30_)]/[c(Glc_0_) + c(Glc_30_)]. AUC_C-Pep0-120_/AUC_Glc0-120_ was calculated, according to the trapezoid method, as ½[½c(C-Pep_0_) + c(C-Pep_30_) + c(C-Pep_60_) + c(C-Pep_90_) + ½c(C-Pep_120_)]/½[½c(Glc_0_) + c(Glc_30_) + c(Glc_60_) + c(Glc_90_) + ½c(Glc_120_)]. Both indices were previously shown to be well-suited to detect genetically limited β-cell function [30]. The acute insulin response (AIR) during the IVGTT was calculated as ½[½c(Ins_0_) + c(Ins_2_) + c(Ins_4_) + c(Ins_6_) + c(Ins_8_) + ½c(Ins_10_)].

### Selection of Tagging SNPs and Genotyping

Based on publicly available HapMap-CEU data (Release #28; http://hapmap.ncbi.nlm.nih.gov/index.html.en) and the tagger function of Haploview 4.2 (http://www.broadinstitute.org.haploview/haploview), we identified 121 SNPs tagging all common SNPs (minor allele frequencies ≥ 0.05) in the *ARNTL*, *ARNTL2*, *CLOCK*, *CRY1*, *CRY2*, *PER1*, *PER2*, *PER3*, and *TIMELESS* loci (gene regions and up to 5 kb of 5’- and 3’-flanking regions) with an r² ≥ 0.8. Information about the genomic localisation of the tagging SNPs is given in S1 Table. For genotyping, DNA was isolated from whole blood using a commercial kit (NucleoSpin, Macherey & Nagel, Düren, Germany). The tagging SNPs were genotyped by mass spectrometry (massARRAY, Sequenom, Hamburg) and the manufacturer’s iPLEX software, and SNPs that resisted massARRAY’s multiplex design were genotyped with TaqMan assays (Applied Biosystems, Foster City, CA, USA). Eleven SNPs were excluded from further analyses due to failure to achieve Hardy-Weinberg equilibrium (p < 0.05). The genotyping results including call rates, minor allele frequencies, and p-values for Hardy-Weinberg equilibrium are given in S1 Table. The 110 tagging SNPs that were followed-up in association analyses covered 76% of common SNPs in *ARNTL*, 79% in *ARNTL2*, 99% in *CLOCK*, 97% in *CRY1*, 100% in *CRY2*, 86% in *PER1*, 83% in *PER2*, 56% in *PER3*, and 100% in *TIMELESS*.

### Human Liver Tissue Collection

A total of 92 German individuals (34 women/58 men; age 62 ± 12 y, BMI 25 ± 4 kg/m²) undergoing liver surgery was included in the present study. The patients were fasted overnight prior to collection of the liver biopsies. The subjects were tested negative for viral hepatitis and had no liver cirrhosis. Liver samples were taken from normal, non-diseased tissue during surgery, immediately frozen in liquid nitrogen, and stored at -80°C.

### Determination of Liver Tissue Triglyceride Content and Estimation of *de novo* Lipogenesis

Liver tissue was homogenized in phosphate-buffered saline containing 1% Triton X-100 using TissueLyser (Qiagen, Hilden, Germany). Triglyceride contents were quantified in the homogenates with the ADVIA 1800 clinical chemistry analyser (Siemens Healthcare Diagnostics, Eschborn, Germany) and are given in percent corresponding to mg/100 mg tissue [31]. To estimate fatty acid biosynthesis, the palmitate-linoleate ratio was calculated as described earlier [32,33]. This *de novo* lipogenesis index represents the ratio of the main product of fatty acid biosynthesis, i.e., palmitate, and the essential fatty acid linoleate originating from dietary lipids. The index was first evaluated under strictly controlled nutritional conditions [32,33] and later validated in individuals on a habitual diet [34,35]. Separation of liver homogenate extracts into five lipid fractions using thin layer chromatography, trans-esterification of the fatty acids, and quantification by gas chromatography with a flame ionization detector was performed as previously described in detail [34,35].

### Determination of Hepatic *CRY2* mRNA Expression by Real-Time Reverse Transcription PCR

Liver tissue was homogenized using TissueLyser (Qiagen), and total RNA was extracted with the RNeasy Tissue Kit from Qiagen according to the manufacturer's instructions. Total RNA treated with RNase-free DNase I was transcribed into cDNA using Avian myoblastosis virus reverse transcriptase and the first-strand cDNA kit from Roche Diagnostics (Mannheim, Germany). PCR (in technical duplicates) was performed on a LightCycler 480 (Roche Diagnostics) using Probes Master and fluorescent probes from the Universal Probe Library (Roche Diagnostics). Primers were designed using the Roche Probe Design 2 software (Roche Diagnosics) and purchased from TIB MOLBIOL (Berlin, Germany). Primer sequences and PCR conditions can be provided upon request. *CRY2* mRNA data were normalised for the housekeeping gene *RPS13* using the ΔΔCt method.

### Statistical Analyses

Hardy-Weinberg equilibrium was tested using χ² test (one degree of freedom). Continuous variables with skewed distribution were log_*e*_-transformed prior to statistical analysis. Multiple linear regression analysis was performed using the least-squares method. In the regression models, the trait of interest (metabolic endpoint) was chosen as outcome variable, the SNP genotype (in the additive inheritance model) as independent variable, and gender, age, BMI, and ISI OGTT as confounding variables as indicated. For analysis of SNP-gender, SNP-BMI, and SNP-glucose interaction effects on the traits of interest, the respective cross effects were tested by multiple linear regression analysis including the appropriate confounding variables. Conditional SNP analyses were performed by testing all SNPs of interest collectively in one multiple linear regression model. Testing all 110 SNPs in parallel, the study-wide significance threshold was set to p < 0.0005 according to Bonferroni correction for multiple comparisons. In the *in vitro* follow-up studies, multiple linear regression analysis was performed, and p-values < 0.05 were considered significant. For all analyses, the statistical software JMP 10.0 (SAS Institute, Cary, NC, USA) was used.

## Results

The clinical characteristics of the overall study population and the subgroups are given in Table 1. Of the 121 tagging SNPs genotyped, 110 SNPs passed our quality criteria, i.e., they were in Hardy-Weinberg equilibrium (p ≥ 0.05) and/or had a minimum call rate of 90% (S1 Table), and thus entered association analysis.

After appropriate adjustment (body fat was adjusted for gender and age; glucose concentrations and insulin sensitivity for gender, age, and BMI; and insulin secretion for gender, age, BMI, and insulin sensitivity), none of the clock SNPs was significantly associated with body fat content, insulin sensitivity, or insulin secretion (S2–S4 Tables). Nor were significant SNP-gender, SNP-body mass, or SNP-glucose interaction effects observed (S5–S7 Tables).

**Fig 1:**
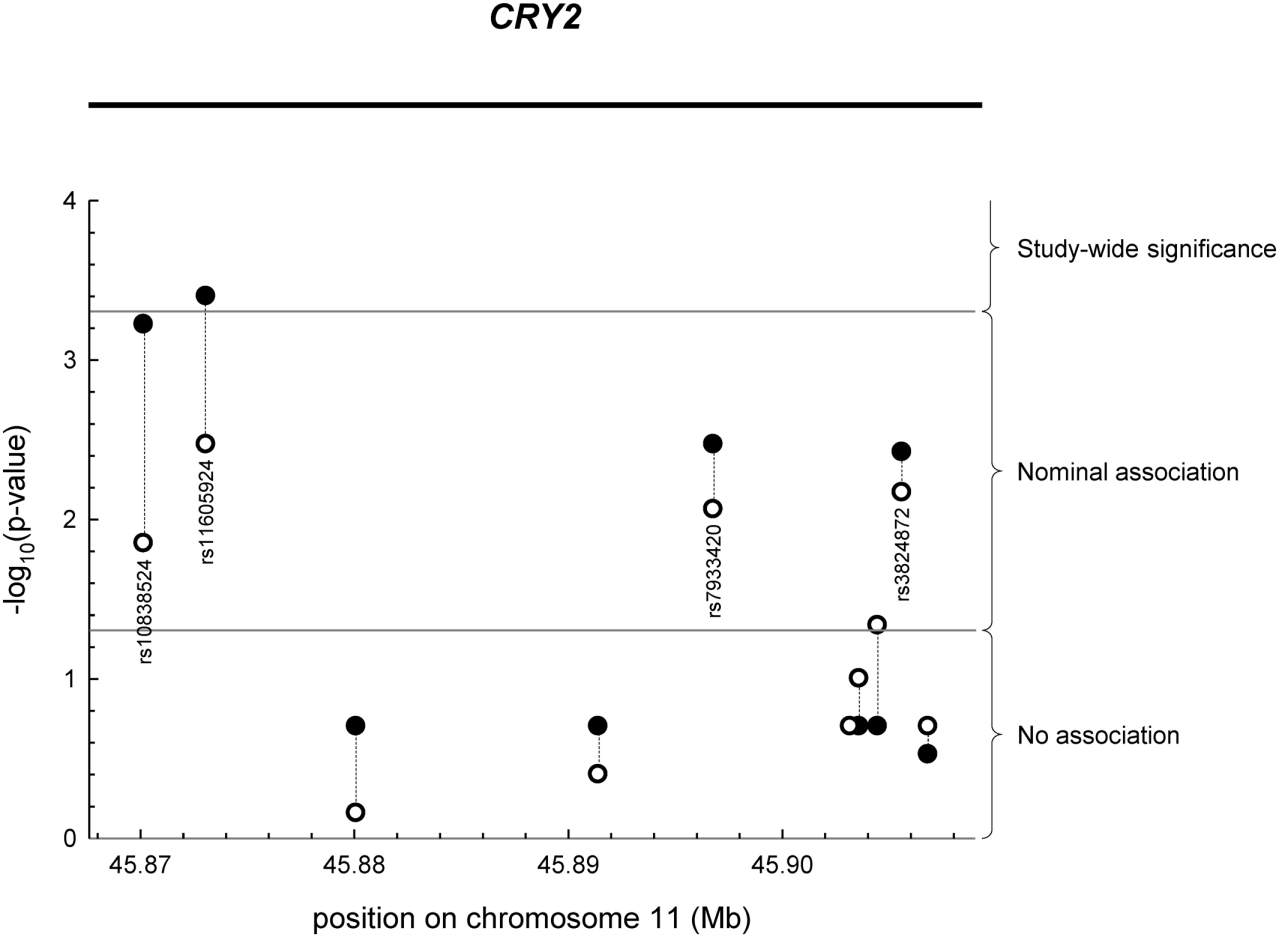
Association of *CRY2* SNPs with fasting glucose concentrations (closed circles) and liver fat content (open circles). SNPs were plotted according to their positions in the *CRY2* locus on chromosome 11 and their association p-values. Study-wide significance p < 0.0005; nominal association 0.0005 ≤ p < 0.05. SNP—single nucleotide polymorphism.

As shown in S2 and S3 Tables and Fig 1, four of the ten *CRY2* SNPs tested were associated with fasting glycaemia (p < 0.004), one of them (rs11605924) reaching study-wide significance (p < 0.0005), and the same SNPs concomitantly revealed nominal associations with liver fat content (p < 0.015). By contrast, the six *CRY2* SNPs that were not associated with fasting glycaemia (p ≥ 0.2) also lacked association with liver fat (p ≥ 0.1; S2 and S3 Tables and Fig 1). This obvious congruence argues against mere chance findings and points to closely linked metabolic effects of CRY2 in liver metabolism. Interestingly, carriers of the minor alleles of the four aforementioned SNPs revealed elevated fasting glycaemia and, concomitantly, reduced liver fat content suggesting that these minor alleles may be involved in redirection of intermediary metabolites from hepatic triglyceride synthesis to gluconeogenesis (S2 and S3 Tables).

Upon interrogation of publicly available data from the Meta-Analyses of Glucose and Insulin-related traits Consortium (MAGIC), three out of the four *CRY2* SNPs, i.e., rs10838524, rs11605924, and rs7933420, revealed very robust effects on fasting glycaemia with congruent effect directions (p < 2.6 x 10^−5^, minor alleles increase fasting glycaemia; [23]). The fourth SNP, i.e., rs3824872, only reached nominal association but again with congruent effect direction (p = 0.0205, minor allele increases fasting glycaemia; [23]). Using conditional analysis of our data, we tested whether the three most robust MAGIC SNPs represent independent signals. Even though incompletely linked (r^2^ < 0.8), as is characteristic of tagging SNPs, none of the SNPs was, independently of the other two SNPs, associated with fasting glycaemia (p > 0.077) or liver fat content (p > 0.3). This points to SNP-SNP interactions in *cis* (direct interactions between DNA regions) or *trans* (interactions mediated by DNA-binding factors) within this locus.

**Fig 2:**
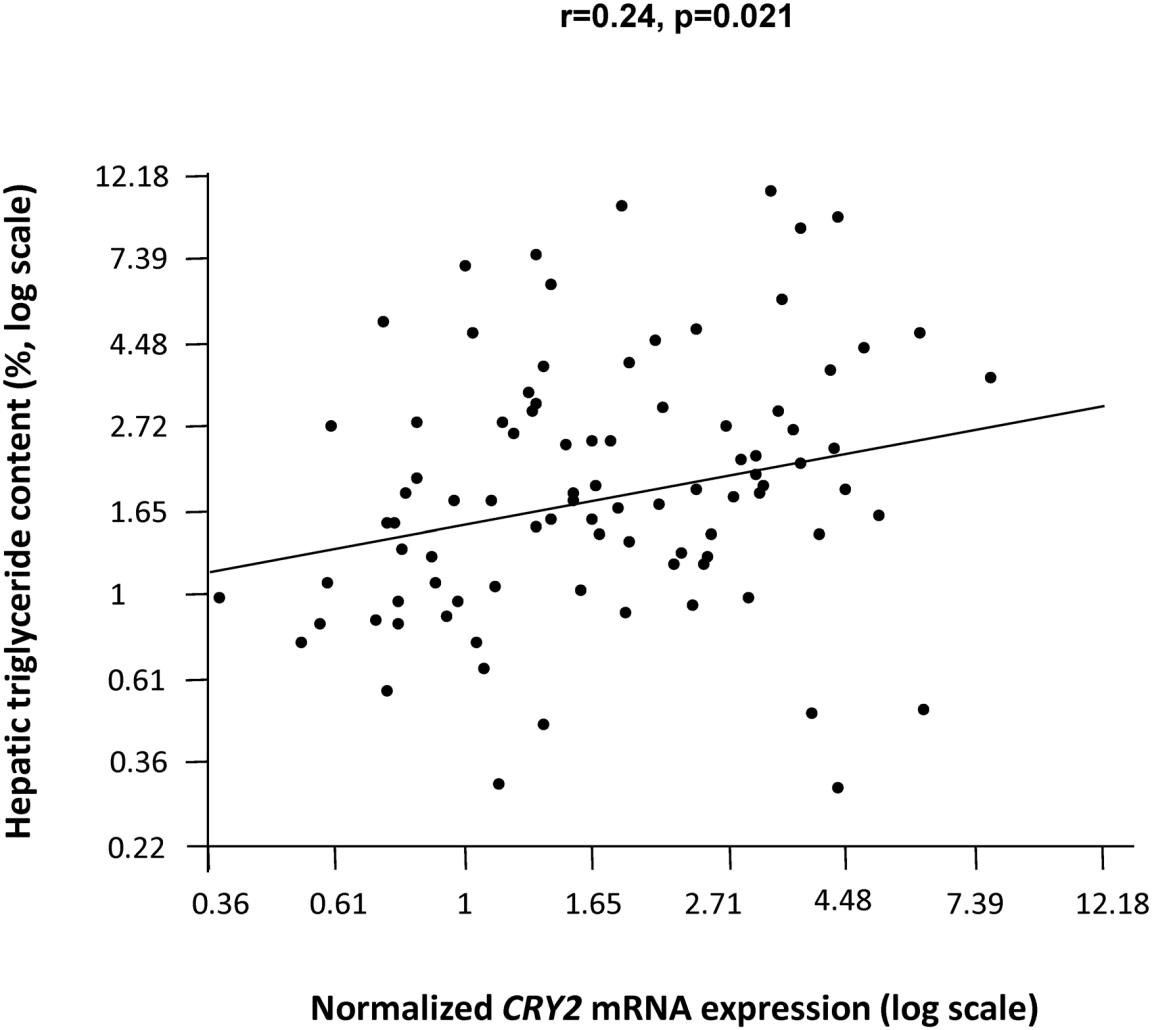
Association of *CRY2* mRNA expression and hepatic triglyceride content. *CRY2* mRNA expression (normalized for the housekeeping gene *RPS13*) and triglyceride contents were measured in a liver tissue collection from 92 patients undergoing liver surgery. Data were log_*e*_-transformed to approximate normal distribution and analysed by multiple linear regression analysis with gender, age, and BMI as covariates.

To further substantiate our liver fat findings, we assessed the relationship between *CRY2* mRNA expression and hepatic triglyceride content in human liver tissue samples characterized by a wide range of triglyceride content (0.3–17.6%). After adjustment for gender, age, and BMI, *CRY2* mRNA expression was directly associated with the tissue triglyceride content (r = 0.24, p = 0.021; Fig 2). To estimate whether CRY2 influences hepatic *de novo* lipogenesis (i.e., fatty acid biosynthesis), we calculated the palmitate-linoleate ratio in the hepatic triglyceride fraction of the liver samples [32,33]. *CRY2* mRNA expression was not significantly associated with the *de novo* lipogenesis index (p = 0.8). This suggests an involvement of CRY2 in re-esterification or lipolysis rather than *de novo* lipogenesis. The sample size of the liver tissue collection was too small to detect reliable associations between the *CRY2* SNPs and hepatic *CRY2* mRNA expression or triglyceride content (data not shown).

## Discussion

Recent meta-analyses of GWA studies revealed fasting glucose-raising variants in *CRY2* which, surprisingly, were not robustly associated with an increased risk for type-2 diabetes [23,24]. In this study, we replicated the effects of four *CRY2* SNPs on fasting glycaemia including their consistent directionality: the minor alleles of all four SNPs were associated with increased fasting glucose concentrations.

More importantly however, we describe herein a novel aspect of the four genetic *CRY2* variants that may explain their lack of association with type-2 diabetes: the fasting glucose-raising alleles were, concomitantly, associated with ~ 30% reduced liver fat content *in vivo*, and liver fat is a well-recognized risk factor for insulin resistance and type-2 diabetes [36,37]. In agreement with this hypothesis, the SNPs were neither associated with insulin sensitivity nor with glucose tolerance (2-h glucose concentrations after oral glucose load). Physiologically, our findings suggest that CRY2 may represent an important molecular switch in hepatic lipid and glucose metabolism favoring triglyceride synthesis at the expense of gluconeogenesis, possibly by channeling intermediary metabolites (e.g., glycerol 3-phosphate) from gluconeogenesis to esterification with fatty acids/triglyceride synthesis. This is strengthened by our finding that CRY2 has no effect on *de novo* lipogenesis/fatty acid biosynthesis.

In accordance with a gluconeogenesis-suppressive effect of CRY2, recently published data points to direct inhibitory interactions of CRY proteins with the glucocorticoid receptor resulting in suppression of glucocorticoid-induced expression of phosphoenolpyruvate carboxykinase 1 [38]. Moreover, Zhang et al. reported that adenoviral CRY1 overexpression in mice inhibits glucagon-stimulated gluconeogenesis through direct interaction with G_s_α and glucagon receptor blockade [39]. Due to their structural relatedness, a similar mechanism is also conceivable for CRY2. Unfortunately, the authors did not assess whether inhibition of gluconeogenesis by CRY1 overexpression is accompanied by an accumulation of hepatic lipids. Based on the glucose-lowering effects of CRY1, the authors speculate that CRY activation may represent an option for anti-diabetic therapy [39]. This idea may be supported by the finding that CRY proteins also limit nuclear factor κB-dependent inflammation, a common hallmark of metabolic diseases [40]. As to CRY2, we think however that CRY activation could be hazardous: our SNP data suggest that any supra-physiological enhancement of CRY2 signaling may favor liver fat accumulation and, thus, promote non-alcoholic fatty liver disease and type-2 diabetes. How CRY2 promotes triglyceride synthesis molecularly is currently unknown and deserves further investigations.

The relevance of our negative findings with respect to the impact of *ARNTL*, *ARNTL2*, *CLOCK*, *CRY1*, *PER1*, *PER2*, *PER3*, and *TIMELESS* on metabolic traits may be questioned due to the limited sample size of our study population (and in particular of our subgroups) that may be sufficiently powered to reveal only rather strong and robust effects, such as those of *CRY2* SNPs on fasting glycaemia. Thus, we cannot exclude that the other clock genes may have modest effects on metabolic traits, and larger studies are needed to address this.

In conclusion, our results support CRY2 as a novel switch in hepatic fuel metabolism promoting triglyceride storage and, concomitantly, limiting glucose production. The anti-steatotic effects of the fasting glucose-raising *CRY2* alleles may explain why these alleles do not increase type-2 diabetes risk. Moreover, if translatable into the prospective setting, this finding may limit the common assumption that increased fasting glycaemia is generally predictive for future type-2 diabetes.

## Supporting Information

S1 Table Genomic localization of the 121 clock SNPs and genotyping results. (DOC)

S2 Table SNP associations with body fat content/distribution. (DOC)

S3 Table SNP associations with glycaemia and insulin sensitivity. (DOC)

S4 Table SNP associations with insulin secretion. (DOC)

S5 Table SNP-gender interaction effects on BMI, body fat content, glucose concentrations, insulin sensitivity, and insulin secretion. (DOC)

S6 Table SNP-BMI interaction effects on glucose concentrations, insulin sensitivity, and insulin secretion. (DOC)

S7 Table SNP-glucose interaction effects on insulin secretion. (DOC)
