# Supplementary material for: Glucose-Raising Polymorphisms in the Human Clock Gene Cryptochrome 2 (CRY2) Affect Hepatic Lipid Content
Source: PLoS One. 2016 Jan 4;11(1):e0145563. doi: 10.1371/journal.pone.0145563 (PMC4699770; doi:10.1371/journal.pone.0145563)
Supplement: S1 Table — (DOC) [file pone.0145563.s001.doc]

**Table S1. Genomic localization of the 121 clock SNPs and genotyping results**

| Gene | SNP | Chromo-some | Chromosomal position | Gene region affected | Call rate (%) | Major/minor allele | MAF | HWE (p-value) |
| --- | --- | --- | --- | --- | --- | --- | --- | --- |
| *ARNTL* | rs7112233 | 11 | 13,255,971 | 5’-flanking region | 99.8 | C/T | 0.22 | 0.8 |
| *ARNTL* | rs7117492 | 11 | 13,257,667 | 5’-flanking region | 99.6 | C/T | 0.22 | 0.3 |
| *ARNTL* | rs12795287 | 11 | 13,260,882 | 5’-flanking region | 99.8 | A/C | 0.47 | 0.3 |
| *ARNTL* | rs11022724 | 11 | 13,272,029 | 5’-flanking region | 99.6 | C/T | 0.14 | 0.7 |
| *ARNTL* | rs2279284 | 11 | 13,298,750 | intron | 99.5 | C/T | 0.27 | 0.8 |
| *ARNTL* | rs7950226 | 11 | 13,318,139 | intron | 99.5 | G/A | 0.47 | 1.0 |
| *ARNTL* | rs10766074 | 11 | 13,318,566 | intron | 99.6 | T/C | 0.16 | 0.2 |
| *ARNTL* | rs4757143 | 11 | 13,329,139 | intron | 99.5 | T/C | 0.14 | 0.5 |
| *ARNTL* | rs4757144 | 11 | 13,331,226 | intron | 99.8 | A/G | 0.42 | 0.3 |
| *ARNTL* | **rs6486121** | 11 | 13,355,770 | intron | 99.6 | T/C | 0.38 | **0.0367** |
| *ARNTL* | rs6486122 | 11 | 13,361,524 | intron | 98.8 | T/C | 0.33 | 0.2 |
| *ARNTL* | rs7937060 | 11 | 13,362,815 | intron | 99.7 | T/C | 0.39 | 0.2 |
| *ARNTL* | rs1562438 | 11 | 13,364,200 | intron | 99.8 | C/T | 0.30 | 0.5 |
| *ARNTL* | **rs1026070** | 11 | 13,364,744 | intron | 99.5 | G/C | 0.05 | **0.0387** |
| *ARNTL* | rs2290036 | 11 | 13,379,788 | intron | 99.8 | T/C | 0.08 | 0.7 |
| *ARNTL* | rs2290037 | 11 | 13,379,973 | splice region | 99.7 | T/C | 0.05 | 0.2 |
| *ARNTL* | rs1868049 | 11 | 13,383,682 | intron | 99.1 | C/T | 0.16 | 0.1 |
| *ARNTL* | rs11022778 | 11 | 13,390,860 | intron | 99.8 | T/G | 0.32 | 0.8 |
| *ARNTL* | rs3816358 | 11 | 13,391,472 | intron | 99.6 | C/A | 0.11 | 0.5 |
| *ARNTL* | rs4757151 | 11 | 13,392,213 | intron | 99.8 | G/A | 0.49 | 0.5 |
| *ARNTL* | rs11600996 | 11 | 13,396,166 | intron | 99.8 | C/T | 0.46 | 0.8 |
| *ARNTL* | rs10766079 | 11 | 13,396,952 | intron | 99.8 | G/A | 0.16 | 0.4 |
| *ARNTL* | rs969485 | 11 | 13,403,043 | intron | 98.3 | A/G | 0.28 | 0.9 |
| *ARNTL* | rs11022783 | 11 | 13,405,899 | intron | 98.9 | G/A | 0.14 | 0.6 |

(continued on next page)

| Gene | SNP | Chromo-some | Chromosomal position | Gene region affected | Call rate (%) | Major/minor allele | MAF | HWE (p-value) |
| --- | --- | --- | --- | --- | --- | --- | --- | --- |
| *ARNTL* | rs10832031 | 11 | 13,406,306 | intron | 99.3 | C/T | 0.15 | 0.7 |
| *ARNTL2* | rs7301841 | 12 | 27,480,524 | 5’-flanking region | 99.5 | G/A | 0.11 | 0.8 |
| *ARNTL2* | rs10842905 | 12 | 27,482,903 | 5’-flanking region | 98.9 | T/C | 0.33 | 0.1 |
| *ARNTL2* | rs7137588 | 12 | 27,483,839 | 5’-flanking region | 99.6 | G/C | 0.35 | 0.7 |
| *ARNTL2* | rs11610949 | 12 | 27,487,468 | intron | 99.9 | T/C | 0.08 | 0.5 |
| *ARNTL2* | rs4964052 | 12 | 27,489,330 | intron | 99.8 | G/T | 0.43 | 0.9 |
| *ARNTL2* | **rs2100840** | 12 | 27,492,738 | intron | 99.7 | G/A | 0.12 | **0.0217** |
| *ARNTL2* | **rs17497606** | 12 | 27,494,449 | intron | 99.5 | C/T | 0.23 | **<0.0001** |
| *ARNTL2* | rs17497683 | 12 | 27,496,606 | intron | 99.8 | G/C | 0.15 | 0.3 |
| *ARNTL2* | rs11048977 | 12 | 27,497,449 | intron | 99.8 | G/A | 0.20 | 0.8 |
| *ARNTL2* | rs11048978 | 12 | 27,497,477 | intron | 98.9 | C/T | 0.46 | 0.8 |
| *ARNTL2* | rs2968756 | 12 | 27,500,454 | intron | 98.9 | A/G | 0.15 | 0.3 |
| *ARNTL2* | rs4964055 | 12 | 27,502,090 | intron | 98.6 | G/T | 0.09 | 0.06 |
| *ARNTL2* | **rs2968758** | 12 | 27,502,955 | intron | 97.3 | T/G | 0.23 | **0.0388** |
| *ARNTL2* | **rs16931921** | 12 | 27,509,371 | intron | 98.9 | A/G | 0.06 | **0.0003** |
| *ARNTL2* | rs12231701 | 12 | 27,515,516 | intron | 99.3 | G/A | 0.11 | 0.6 |
| *ARNTL2* | rs7306410 | 12 | 27,525,596 | intron | 97.3 | T/C | 0.19 | 0.5 |
| *ARNTL2* | rs4964059 | 12 | 27,529,219 | intron | 99.6 | A/C | 0.35 | 0.2 |
| *ARNTL2* | rs11048994 | 12 | 27,530,778 | intron | 99.6 | G/A | 0.20 | 0.7 |
| *ARNTL2* | rs11048995 | 12 | 27,531,435 | intron | 99.3 | A/G | 0.45 | 0.1 |
| *ARNTL2* | rs7304939 | 12 | 27,544,345 | intron | 99.4 | C/T | 0.09 | 0.6 |
| *ARNTL2* | rs11048997 | 12 | 27,544,990 | intron | 99.8 | T/C | 0.05 | 0.9 |
| *ARNTL2* | rs12319133 | 12 | 27,549,082 | intron | 99.7 | G/C | 0.11 | 0.7 |
| *ARNTL2* | **rs4488298** | 12 | 27,550,577 | intron | 99.0 | G/A | 0.09 | **0.0278** |

(continued on next page)

| Gene | SNP | Chromo-some | Chromosomal position | Gene region affected | Call rate (%) | Major/minor allele | MAF | HWE (p-value) |
| --- | --- | --- | --- | --- | --- | --- | --- | --- |
| *ARNTL2* | rs4409932 | 12 | 27,554,699 | intron | 99.8 | G/A | 0.16 | 0.8 |
| *ARNTL2* | rs2306074 | 12 | 27,555,786 | intron | 99.5 | T/C | 0.29 | 0.5 |
| *ARNTL2* | rs4931075 | 12 | 27,565,146 | intron | 99.8 | G/A | 0.17 | 0.4 |
| *ARNTL2* | rs11049004 | 12 | 27,567,836 | intron | 99.8 | C/G | 0.08 | 0.4 |
| *ARNTL2* | rs2682706 | 12 | 27,569,179 | intron | 99.5 | T/C | 0.22 | 0.7 |
| *CLOCK* | rs10462028 | 4 | 56,298,300 | 3’-untranslated region | 99.9 | G/A | 0.33 | 0.2 |
| *CLOCK* | rs1801260 | 4 | 56,301,369 | 3’-untranslated region | 99.7 | A/G | 0.28 | 0.6 |
| *CLOCK* | rs3792603 | 4 | 56,302,058 | intron | 99.6 | A/G | 0.21 | 0.8 |
| *CLOCK* | rs17777927 | 4 | 56,312,633 | intron | 99.8 | C/G | 0.06 | 0.3 |
| *CLOCK* | rs4864996 | 4 | 56,319,088 | intron | 99.4 | A/C | 0.30 | 0.8 |
| *CLOCK* | rs11725422 | 4 | 56,321,703 | intron | 99.9 | G/A | 0.05 | 0.5 |
| *CLOCK* | rs1554483 | 4 | 56,321,817 | intron | 99.8 | C/G | 0.34 | 0.2 |
| *CLOCK* | rs11932595 | 4 | 56,323,597 | intron | 99.8 | A/G | 0.40 | 0.6 |
| *CLOCK* | rs1522113 | 4 | 56,331,771 | intron | 98.9 | G/A | 0.05 | 0.5 |
| *CLOCK* | rs11733959 | 4 | 56,337,164 | intron | 99.8 | A/G | 0.05 | 0.5 |
| *CLOCK* | rs6554281 | 4 | 56,377,898 | intron | 99.7 | T/C | 0.07 | 0.4 |
| *CLOCK* | **rs6850524** | 4 | 56,381,997 | intron | **82.9** | G/C | 0.41 | **<0.0001** |
| *CLOCK* | rs4864548 | 4 | 56,413,803 | 5’-flanking region | 99.7 | G/A | 0.35 | 0.9 |
| *CLOCK* | rs1979604 | 4 | 56,418,755 | 5’-flanking region | 99.8 | C/A | 0.28 | 0.6 |
| *CLOCK* | rs726967 | 4 | 56,421,713 | 5’-flanking region | 98.8 | A/T | 0.37 | 0.4 |
| *CRY1* | rs10861688 | 12 | 107,394,048 | intron | 98.9 | C/T | 0.18 | 0.9 |
| *CRY1* | rs12368868 | 12 | 107,405,591 | intron | 99.8 | A/G | 0.09 | 0.7 |
| *CRY1* | rs1921126 | 12 | 107,411,605 | intron | 100 | T/C | 0.44 | 0.06 |
| *CRY1* | **rs10746075** | 12 | 107,423,983 | intron | 99.8 | T/A | 0.42 | **0.0316** |

(continued on next page)

| Gene | SNP | Chromo-some | Chromosomal position | Gene region affected | Call rate (%) | Major/minor allele | MAF | HWE (p-value) |
| --- | --- | --- | --- | --- | --- | --- | --- | --- |
| *CRY1* | rs11113179 | 12 | 107,452,785 | intron | 98.2 | C/T | 0.09 | 0.3 |
| *CRY1* | rs11113181 | 12 | 107,468,261 | intron | 99.9 | A/G | 0.17 | 1.0 |
| *CRY1* | rs17289712 | 12 | 107,468,968 | intron | 99.3 | A/G | 0.04 | 0.2 |
| *CRY2* | rs10838524 | 11 | 45,870,177 | intron | 99.5 | G/A | 0.45 | 0.7 |
| *CRY2* | rs11605924 | 11 | 45,873,091 | intron | 99.4 | C/A | 0.48 | 0.8 |
| *CRY2* | rs1401417 | 11 | 45,880,110 | intron | 98.8 | C/G | 0.24 | 0.5 |
| *CRY2* | rs7123390 | 11 | 45,891,418 | intron | 99.8 | G/A | 0.27 | 0.8 |
| *CRY2* | rs7933420 | 11 | 45,896,797 | intron | 97.9 | T/A | 0.50 | 0.6 |
| *CRY2* | rs10838527 | 11 | 45,903,194 | 3’-untranslated region | 98.9 | A/G | 0.08 | 0.2 |
| *CRY2* | rs2292910 | 11 | 45,903,613 | 3’-untranslated region | 99.9 | C/A | 0.34 | 0.8 |
| *CRY2* | rs6798 | 11 | 45,904,477 | 3’-untranslated region | 99.2 | C/T | 0.10 | 0.4 |
| *CRY2* | rs3824872 | 11 | 45,905,605 | 3’-flanking region | 99.9 | C/A | 0.20 | 1.0 |
| *CRY2* | rs1554338 | 11 | 45,906,830 | 3’-flanking region | 99.8 | A/G | 0.06 | 0.5 |
| *PER1* | rs9914077 | 17 | 8,040,294 | 3’-flanking region | 99.9 | A/G | 0.42 | 0.7 |
| *PER1* | rs2289591 | 17 | 8,048,010 | intron | 99.2 | C/A | 0.25 | 0.07 |
| *PER1* | rs2735611 | 17 | 8,048,283 | coding region (G749G) | 97.7 | A/G | 0.17 | 0.8 |
| *PER1* | rs3027188 | 17 | 8,048,985 | intron | 99.9 | C/G | 0.16 | 0.6 |
| *PER1* | rs2304911 | 17 | 8,050,979 | intron | 99.8 | A/G | 0.05 | 0.3 |
| *PER1* | rs2518023 | 17 | 8,056,606 | 5’-flanking region | 99.9 | G/T | 0.10 | 0.6 |
| *PER2* | rs881933 | 2 | 239,151,041 | 3’-flanking region | 99.9 | G/C | 0.33 | 0.2 |
| *PER2* | rs934945 | 2 | 239,155,053 | coding region (G1244E) | 99.8 | C/T | 0.20 | 0.3 |
| *PER2* | rs2304670 | 2 | 239,165,636 | coding region (A664A) | 98.9 | C/T | 0.09 | 0.5 |
| *PER2* | rs2304669 | 2 | 239,165,663 | coding region (A655A) | 99.8 | T/C | 0.14 | 0.8 |
| *PER2* | rs7570188 | 2 | 239,172,863 | intron | 99.7 | T/G | 0.06 | 0.2 |

(continued on next page)

| Gene | SNP | Chromo-some | Chromosomal position | Gene region affected | Call rate (%) | Major/minor allele | MAF | HWE (p-value) |
| --- | --- | --- | --- | --- | --- | --- | --- | --- |
| *PER2* | rs3739064 | 2 | 239,176,386 | intron | 99.9 | A/G | 0.23 | 0.4 |
| *PER2* | rs11894535 | 2 | 239,177,073 | intron | 98.2 | C/T | 0.22 | 0.2 |
| *PER2* | rs10462023 | 2 | 239,184,581 | intron | 99.9 | G/A | 0.34 | 0.1 |
| *PER2* | rs2304673 | 2 | 239,185,933 | intron | 99.3 | T/G | 0.14 | 0.8 |
| *PER2* | rs11892306 | 2 | 239,188,479 | intron | 99.9 | G/T | 0.30 | 0.6 |
| *PER2* | rs11894491 | 2 | 239,198,325 | 5’-flanking region | 99.9 | G/A | 0.35 | 0.6 |
| *PER3* | rs875994 | 1 | 7,852,919 | intron | 99.5 | T/C | 0.16 | 0.4 |
| *PER3* | rs228682 | 1 | 7,856,346 | intron | 99.4 | T/C | 0.42 | 0.8 |
| *PER3* | rs228666 | 1 | 7,868,725 | intron | 99.5 | T/C | 0.32 | 1.0 |
| *PER3* | **rs228669** | 1 | 7,870,048 | coding region (S445S) | 99.5 | C/T | 0.07 | **0.0093** |
| *PER3* | rs1891217 | 1 | 7,871,849 | intron | 99.6 | C/T | 0.09 | 0.8 |
| *PER3* | rs2172563 | 1 | 7,874,043 | intron | 99.5 | G/A | 0.22 | 0.9 |
| *PER3* | rs12061787 | 1 | 7,888,730 | intron | 99.5 | G/C | 0.14 | 0.08 |
| *PER3* | rs2640908 | 1 | 7,889,941 | coding region (T969T) | 99.5 | C/T | 0.20 | 0.1 |
| *PER3* | **rs228665** | 1 | 7,890,956 | intron | 99.5 | C/G | 0.34 | **0.0179** |
| *PER3* | rs228675 | 1 | 7,900,395 | intron | 99.5 | A/T | 0.32 | 0.8 |
| *TIMELESS* | rs17441402 | 12 | 56,807,841 | 3’-flanking region | 99.8 | A/T | 0.13 | 0.6 |
| *TIMELESS* | rs4759206 | 12 | 56,808,264 | 3’-flanking region | 98.0 | G/A | 0.49 | 0.09 |
| *TIMELESS* | rs2291738 | 12 | 56,815,281 | splice region | 99.0 | T/C | 0.47 | 0.3 |
| *TIMELESS* | rs774049 | 12 | 56,816,978 | intron | 99.9 | C/T | 0.04 | 0.2 |
| *TIMELESS* | rs774035 | 12 | 56,825,749 | intron | 98.0 | A/G | 0.46 | 0.8 |
| *TIMELESS* | rs11171846 | 12 | 56,828,056 | intron | 99.5 | C/T | 0.10 | 0.7 |
| *TIMELESS* | rs11171852 | 12 | 56,833,224 | intron | 99.9 | T/C | 0.48 | 0.9 |
| *TIMELESS* | rs4630333 | 12 | 56,837,416 | intron | 98.2 | C/T | 0.42 | 0.7 |

(continued on next page)

| Gene | SNP | Chromo-some | Chromosomal position | Gene region affected | Call rate (%) | Major/minor allele | MAF | HWE (p-value) |
| --- | --- | --- | --- | --- | --- | --- | --- | --- |
| *TIMELESS* | rs774044 | 12 | 56,837,979 | intron | 100 | C/T | 0.06 | 0.6 |

Chromosomal position data are Ensembl data. SNPs excluded from association analyses due to a call rate below 90% or failure to achieve Hardy-Weinberg equilibrium (p<0.05) are marked in bold. HWE – Hardy-Weinberg equilibrium; MAF – minor allele frequency; SNP – single nucleotide polymorphism
