# Supplementary material for: Glucose-Raising Polymorphisms in the Human Clock Gene Cryptochrome 2 (CRY2) Affect Hepatic Lipid Content
Source: PLoS One. 2016 Jan 4;11(1):e0145563. doi: 10.1371/journal.pone.0145563 (PMC4699770; doi:10.1371/journal.pone.0145563)
Supplement: S4 Table — (DOC) [file pone.0145563.s004.doc]

**Table S4. SNP associations with insulin secretion** (OGTT: N=1,632; IVGTT: N=314)

| Gene | SNP | AUC_Ins0-30_/AUC_Glc0-30_ (OGTT) | AUC_C-Pep0-120_/AUC_Glc0-120_ (OGTT) | AIR (IVGTT) |
| --- | --- | --- | --- | --- |
| *ARNTL* | rs7112233 | 0.8 (0.005) | 0.7 (-0.008) | 0.2 (0.063) |
| *ARNTL* | rs7117492 | 0.8 (0.004) | 0.9 (-0.003) | 0.1 (-0.079) |
| *ARNTL* | rs12795287 | 0.8 (0.004) | 0.4 (-0.018) | 0.8 (-0.011) |
| *ARNTL* | rs11022724 | 0.9 (-0.001) | 0.5 (0.013) | 0.3 (-0.053) |
| *ARNTL* | rs2279284 | 0.5 (0.012) | 1.0 (0.001) | 0.1 (0.078) |
| *ARNTL* | rs7950226 | 0.5 (0.009) | 0.5 (0.015) | 0.2 (0.062) |
| *ARNTL* | rs10766074 | 0.3 (-0.016) | 0.2 (-0.028) | 0.8 (0.013) |
| *ARNTL* | rs4757143 | 0.9 (-0.003) | 0.4 (0.018) | 0.2 (-0.059) |
| *ARNTL* | rs4757144 | 0.8 (0.004) | 0.9 (0.002) | 0.9 (-0.004) |
| *ARNTL* | rs6486122 | **0.0067** (0.042) | 0.1 (0.034) | 0.2 (0.061) |
| *ARNTL* | rs7937060 | **0.0171** (0.037) | 0.1 (0.035) | 0.6 (0.022) |
| *ARNTL* | rs1562438 | 0.1 (0.024) | 0.8 (0.007) | 0.3 (0.047) |
| *ARNTL* | rs2290036 | 0.3 (-0.017) | 0.8 (0.006) | 0.3 (-0.048) |
| *ARNTL* | rs2290037 | **0.0292** (-0.034) | 0.9 (0.002) | 0.7 (-0.021) |
| *ARNTL* | rs1868049 | 0.9 (0.002) | 0.5 (-0.014) | 0.8 (-0.014) |
| *ARNTL* | rs11022778 | 0.4 (-0.012) | 0.8 (0.007) | 0.6 (0.025) |
| *ARNTL* | rs3816358 | 0.6 (0.008) | 0.3 (0.024) | 0.9 (0.006) |
| *ARNTL* | rs4757151 | 0.2 (0.019) | 0.6 (0.010) | 0.9 (-0.004) |
| *ARNTL* | rs11600996 | 0.06 (-0.029) | 0.5 (-0.015) | 0.5 (0.030) |
| *ARNTL* | rs10766079 | 0.8 (-0.005) | 0.9 (-0.002) | 0.5 (0.036) |
| *ARNTL* | rs969485 | 0.08 (-0.027) | 0.2 (-0.031) | 0.3 (0.053) |
| *ARNTL* | rs11022783 | 0.6 (-0.008) | 0.5 (-0.013) | 0.9 (0.008) |
| *ARNTL* | rs10832031 | 0.7 (-0.007) | 0.4 (0.018) | 0.8 (-0.011) |

(continued on next page)

| Gene | SNP | AUC_Ins0-30_/AUC_Glc0-30_ (OGTT) | AUC_C-Pep0-120_/AUC_Glc0-120_ (OGTT) | AIR (IVGTT) |
| --- | --- | --- | --- | --- |
| *ARNTL2* | rs7301841 | 0.4 (0.013) | 0.1 (0.032) | 0.2 (0.064) |
| *ARNTL2* | rs10842905 | 0.8 (0.005) | 0.8 (0.006) | 0.9 (0.008) |
| *ARNTL2* | rs7137588 | 0.3 (0.016) | 1.0 (0.001) | 0.2 (0.068) |
| *ARNTL2* | rs11610949 | 0.8 (0.004) | 0.3 (-0.020) | 0.2 (0.065) |
| *ARNTL2* | rs4964052 | 0.9 (0.002) | 0.3 (0.022) | 0.8 (0.014) |
| *ARNTL2* | rs17497683 | 0.6 (0.007) | 0.7 (-0.009) | 0.6 (-0.028) |
| *ARNTL2* | rs11048977 | 0.8 (0.004) | 0.4 (0.019) | 0.6 (-0.024) |
| *ARNTL2* | rs11048978 | 0.8 (-0.004) | 0.2 (0.025) | 0.5 (0.033) |
| *ARNTL2* | rs2968756 | 0.7 (-0.006) | 0.6 (-0.013) | **0.0190** (-0.115) |
| *ARNTL2* | rs4964055 | 0.4 (-0.012) | **0.0232** (-0.048) | 0.5 (0.037) |
| *ARNTL2* | rs12231701 | 0.5 (0.011) | 0.2 (0.027) | 0.2 (0.057) |
| *ARNTL2* | rs7306410 | 0.4 (0.012) | 0.8 (0.006) | 0.07 (0.089) |
| *ARNTL2* | rs4964059 | 0.6 (0.008) | 0.7 (-0.008) | 0.4 (0.042) |
| *ARNTL2* | rs11048994 | 0.09 (0.026) | 0.05 (0.041) | 0.2 (-0.066) |
| *ARNTL2* | rs11048995 | 0.6 (0.008) | 0.8 (-0.006) | 0.5 (-0.034) |
| *ARNTL2* | rs7304939 | 0.8 (-0.005) | 0.6 (0.011) | 0.9 (-0.004) |
| *ARNTL2* | rs11048997 | 0.9 (0.001) | 1.0 (0.001) | 0.8 (0.011) |
| *ARNTL2* | rs12319133 | 0.8 (0.004) | 0.8 (0.006) | 0.08 (0.085) |
| *ARNTL2* | rs4409932 | 0.9 (-0.002) | 0.6 (0.010) | 0.2 (0.065) |
| *ARNTL2* | rs2306074 | 0.7 (-0.007) | 0.7 (-0.009) | 0.9 (0.006) |
| *ARNTL2* | rs4931075 | 0.4 (0.012) | 1.0 (-0.000) | 0.6 (-0.027) |
| *ARNTL2* | rs11049004 | 0.3 (-0.015) | 0.5 (-0.013) | 0.1 (0.075) |
| *ARNTL2* | rs2682706 | 0.6 (0.008) | 0.7 (0.009) | 0.4 (-0.039) |
| *CLOCK* | rs10462028 | 0.4 (0.013) | 0.6 (0.011) | 0.9 (0.007) |
| *CLOCK* | rs1801260 | 0.5 (0.011) | 0.9 (0.003) | 0.7 (-0.022) |

(continued on next page)

| Gene | SNP | AUC_Ins0-30_/AUC_Glc0-30_ (OGTT) | AUC_C-Pep0-120_/AUC_Glc0-120_ (OGTT) | AIR (IVGTT) |
| --- | --- | --- | --- | --- |
| *CLOCK* | rs3792603 | 0.7 (0.006) | 1.0 (-0.000) | 0.5 (-0.032) |
| *CLOCK* | rs17777927 | 0.5 (0.011) | 0.5 (0.015) | 1.0 (-0.001) |
| *CLOCK* | rs4864996 | 0.7 (-0.007) | 0.7 (-0.009) | 0.4 (0.040) |
| *CLOCK* | rs11725422 | 0.6 (0.007) | 0.4 (0.018) | 0.2 (0.056) |
| *CLOCK* | rs1554483 | 0.4 (-0.014) | 0.5 (-0.015) | 0.3 (0.053) |
| *CLOCK* | rs11932595 | 0.8 (-0.004) | 0.5 (-0.014) | 0.5 (-0.031) |
| *CLOCK* | rs1522113 | 0.6 (0.008) | 0.4 (0.019) | 0.3 (0.056) |
| *CLOCK* | rs11733959 | 0.6 (0.007) | 0.4 (0.019) | 0.2 (0.058) |
| *CLOCK* | rs6554281 | 0.5 (-0.011) | 0.9 (-0.003) | 0.4 (-0.038) |
| *CLOCK* | rs4864548 | 0.7 (-0.006) | 0.9 (-0.004) | 0.2 (0.063) |
| *CLOCK* | rs1979604 | 0.5 (0.011) | 0.9 (0.002) | 0.7 (-0.022) |
| *CLOCK* | rs726967 | 0.6 (-0.007) | 1.0 (-0.000) | 0.3 (-0.047) |
| *CRY1* | rs10861688 | 0.2 (0.020) | 0.2 (0.025) | 0.8 (0.012) |
| *CRY1* | rs12368868 | 0.4 (-0.012) | 0.2 (-0.025) | 0.3 (-0.052) |
| *CRY1* | rs1921126 | 0.1 (-0.023) | 0.2 (-0.028) | 0.6 (-0.026) |
| *CRY1* | rs11113179 | 0.6 (-0.008) | 0.4 (-0.018) | 0.3 (-0.052) |
| *CRY1* | rs11113181 | 0.3 (0.018) | 0.3 (0.024) | 1.0 (-0.003) |
| *CRY1* | rs17289712 | 0.2 (-0.019) | 0.06 (-0.039) | 0.1 (-0.081) |
| *CRY2* | rs10838524 | 0.5 (-0.011) | 0.2 (-0.029) | 0.4 (0.040) |
| *CRY2* | rs11605924 | 0.6 (-0.009) | 0.2 (-0.026) | 0.5 (0.034) |
| *CRY2* | rs1401417 | 0.5 (0.010) | 1.0 (-0.001) | 0.5 (0.032) |
| *CRY2* | rs7123390 | 0.3 (0.015) | 0.9 (0.002) | 0.6 (0.029) |
| *CRY2* | rs7933420 | 0.5 (-0.010) | 0.2 (-0.026) | 0.5 (0.034) |
| *CRY2* | rs10838527 | 0.6 (-0.009) | 0.5 (-0.014) | 0.5 (0.037) |
| *CRY2* | rs2292910 | 0.8 (0.005) | 0.4 (-0.016) | 0.7 (0.017) |

(continued on next page)

| Gene | SNP | AUC_Ins0-30_/AUC_Glc0-30_ (OGTT) | AUC_C-Pep0-120_/AUC_Glc0-120_ (OGTT) | AIR (IVGTT) |
| --- | --- | --- | --- | --- |
| *CRY2* | rs6798 | 0.1 (-0.023) | 0.08 (-0.037) | 0.6 (0.025) |
| *CRY2* | rs3824872 | 0.08 (-0.027) | 0.1 (-0.034) | 0.6 (0.026) |
| *CRY2* | rs1554338 | 0.9 (0.003) | 0.5 (0.015) | 0.3 (-0.050) |
| *PER1* | rs9914077 | **0.0280** (0.034) | 0.3 (0.022) | 0.5 (-0.035) |
| *PER1* | rs2289591 | 0.2 (-0.021) | 0.3 (-0.022) | 0.6 (-0.027) |
| *PER1* | rs2735611 | 0.1 (-0.024) | 0.2 (-0.028) | 0.5 (-0.030) |
| *PER1* | rs3027188 | 0.2 (-0.019) | 0.5 (-0.015) | 0.8 (0.013) |
| *PER1* | rs2304911 | 0.5 (-0.010) | 0.7 (-0.009) | 0.4 (0.040) |
| *PER1* | rs2518023 | 0.5 (-0.010) | 0.6 (-0.011) | 0.7 (-0.017) |
| *PER2* | rs881933 | **0.0063** (-0.042) | 0.6 (-0.011) | 0.7 (-0.021) |
| *PER2* | rs934945 | **0.0371** (-0.032) | 0.9 (0.002) | 0.4 (-0.045) |
| *PER2* | rs2304670 | 0.9 (-0.002) | 0.4 (-0.017) | 0.7 (-0.017) |
| *PER2* | rs2304669 | 0.4 (-0.014) | 0.6 (-0.010) | 0.9 (0.006) |
| *PER2* | rs7570188 | 0.5 (0.011) | 0.8 (0.005) | 0.9 (0.004) |
| *PER2* | rs3739064 | 0.6 (-0.008) | 0.3 (-0.020) | 0.9 (-0.008) |
| *PER2* | rs11894535 | 0.5 (0.011) | 0.5 (0.013) | 0.5 (0.030) |
| *PER2* | rs10462023 | 0.2 (0.018) | 0.3 (0.021) | 0.6 (0.027) |
| *PER2* | rs2304673 | 0.5 (-0.011) | 0.5 (-0.013) | 0.6 (0.026) |
| *PER2* | rs11892306 | 1.0 (0.000) | 0.6 (-0.011) | 1.0 (0.000) |
| *PER2* | rs11894491 | 0.1 (-0.024) | 0.5 (-0.014) | 0.7 (-0.018) |
| *PER3* | rs875994 | **0.0467** (0.031) | 0.2 (0.028) | 0.7 (0.017) |
| *PER3* | rs228682 | 0.6 (-0.008) | 0.5 (-0.013) | 0.8 (-0.014) |
| *PER3* | rs228666 | 0.06 (0.029) | 0.2 (0.026) | 0.1 (0.078) |
| *PER3* | rs1891217 | 0.5 (-0.010) | 0.2 (-0.029) | 0.1 (-0.080) |
| *PER3* | rs2172563 | 0.5 (-0.010) | 0.8 (-0.006) | 0.6 (-0.024) |

(continued on next page)

| Gene | SNP | AUC_Ins0-30_/AUC_Glc0-30_ (OGTT) | AUC_C-Pep0-120_/AUC_Glc0-120_ (OGTT) | AIR (IVGTT) |
| --- | --- | --- | --- | --- |
| *PER3* | rs12061787 | 0.2 (0.018) | 0.7 (0.007) | 0.9 (-0.005) |
| *PER3* | rs2640908 | 0.2 (-0.019) | 1.0 (0.001) | 0.8 (-0.012) |
| *PER3* | rs228675 | 0.06 (0.029) | 0.2 (0.026) | 0.1 (0.078) |
| *TIMELESS* | rs17441402 | 0.1 (0.022) | 0.7 (-0.007) | 0.8 (0.010) |
| *TIMELESS* | rs4759206 | 0.2 (0.020) | 0.3 (0.023) | 0.4 (0.042) |
| *TIMELESS* | rs2291738 | 0.8 (0.005) | 0.3 (-0.023) | 0.7 (0.019) |
| *TIMELESS* | rs774049 | 0.5 (-0.011) | 1.0 (-0.000) | 0.2 (-0.067) |
| *TIMELESS* | rs774035 | 0.9 (0.003) | 0.4 (0.017) | 0.6 (-0.024) |
| *TIMELESS* | rs11171846 | 0.8 (0.004) | 0.4 (0.019) | 0.4 (-0.040) |
| *TIMELESS* | rs11171852 | 0.7 (-0.007) | 0.7 (-0.007) | 0.5 (-0.031) |
| *TIMELESS* | rs4630333 | 0.9 (0.002) | 0.5 (0.016) | 0.6 (-0.024) |
| *TIMELESS* | rs774044 | 0.2 (-0.020) | 0.06 (-0.040) | 0.3 (-0.050) |

Data represent p-values and, in brackets, effect sizes of the minor alleles (standardized β). Association between SNP genotype (additive inheritance model) and insulin secretion was tested by multiple linear regression analysis (standard least squares method) with gender, age, BMI, and (OGTT-derived) insulin sensitivity as covariates. Nominal associations (p<0.05) are marked by using bold fonts. AIR – acute insulin response; AUC – area under the curve; C-Pep – C-peptide; Glc – glucose; Ins – insulin; IVGTT – intravenous glucose tolerance test; OGTT – oral glucose tolerance test; SNP – single nucleotide polymorphism
