# Supplementary material for: Glucose-Raising Polymorphisms in the Human Clock Gene Cryptochrome 2 (CRY2) Affect Hepatic Lipid Content
Source: PLoS One. 2016 Jan 4;11(1):e0145563. doi: 10.1371/journal.pone.0145563 (PMC4699770; doi:10.1371/journal.pone.0145563)
Supplement: S5 Table — (DOC) [file pone.0145563.s005.doc]

**Table S5. SNP-gender interaction effects on BMI, body fat content, glucose concentrations, insulin sensitivity, and insulin secretion**

| Gene | SNP | BMI | Body fat content | Glucose (fasting) | 2-h Glucose | ISI (OGTT) | AUC_Ins0-30_/ AUC_Glc0-30_ | AUC_C-Pep0-120_/ AUC_Glc0-120_ |
| --- | --- | --- | --- | --- | --- | --- | --- | --- |
| *ARNTL* | rs7112233 | **0.0170** | **0.0049** | **0.0156** | 0.5 | 0.2 | 0.5 | 0.7 |
| *ARNTL* | rs7117492 | 0.9 | 0.1 | 0.2 | 1.0 | **0.0110** | 1.0 | 0.3 |
| *ARNTL* | rs12795287 | 0.07 | 0.3 | 0.9 | 0.7 | 0.2 | 0.9 | 0.7 |
| *ARNTL* | rs11022724 | 0.5 | 0.6 | 0.5 | 0.9 | 0.09 | 1.0 | 0.7 |
| *ARNTL* | rs2279284 | 0.3 | 0.6 | 0.09 | 0.5 | 0.1 | 0.9 | 0.6 |
| *ARNTL* | rs7950226 | 0.5 | 0.8 | 0.7 | 0.07 | 0.7 | 0.3 | 0.3 |
| *ARNTL* | rs10766074 | 0.9 | 0.7 | 0.5 | 0.4 | 0.5 | 0.2 | 0.6 |
| *ARNTL* | rs4757143 | 1.0 | 0.9 | 0.4 | 0.8 | 0.08 | 0.7 | 0.4 |
| *ARNTL* | rs4757144 | 1.0 | 0.8 | 0.7 | 0.9 | 1.0 | 0.3 | 0.2 |
| *ARNTL* | rs6486122 | 1.0 | 0.9 | 0.5 | 0.4 | 0.8 | 0.3 | 0.4 |
| *ARNTL* | rs7937060 | 1.0 | 0.6 | 0.9 | 0.3 | 0.8 | 0.4 | 0.1 |
| *ARNTL* | rs1562438 | 0.4 | 0.9 | 0.9 | 0.7 | 0.5 | 0.4 | 0.3 |
| *ARNTL* | rs2290036 | 0.6 | 1.0 | 0.9 | 0.8 | 0.3 | 0.9 | 0.6 |
| *ARNTL* | rs2290037 | 0.9 | 0.9 | 1.0 | 0.6 | 0.6 | 0.6 | 0.7 |
| *ARNTL* | rs1868049 | 0.8 | 0.3 | 0.2 | 0.9 | 0.5 | 0.5 | 0.6 |
| *ARNTL* | rs11022778 | 0.6 | 0.4 | **0.0155** | **0.0052** | 1.0 | **0.0346** | **0.0405** |
| *ARNTL* | rs3816358 | 0.2 | 0.5 | 0.2 | 0.3 | 0.9 | 0.4 | 0.4 |
| *ARNTL* | rs4757151 | 0.6 | 0.7 | 0.9 | 0.4 | 0.8 | 0.3 | 0.6 |
| *ARNTL* | rs11600996 | 0.9 | 0.5 | 0.6 | 0.3 | 0.1 | 1.0 | 0.1 |
| *ARNTL* | rs10766079 | 0.9 | 0.5 | 0.6 | 0.5 | 0.7 | 0.4 | 0.2 |
| *ARNTL* | rs969485 | 0.9 | 0.5 | 0.5 | 0.4 | 0.3 | 0.9 | 0.3 |
| *ARNTL* | rs11022783 | 0.8 | 0.4 | 0.7 | 0.9 | 0.9 | 0.4 | 0.3 |
| *ARNTL* | rs10832031 | 0.9 | 0.8 | 0.2 | 0.8 | 0.4 | 0.8 | 0.5 |

(continued on next page)

| Gene | SNP | BMI | Body fat content | Glucose (fasting) | 2-h Glucose | ISI (OGTT) | AUC_Ins0-30_/ AUC_Glc0-30_ | AUC_C-Pep0-120_/ AUC_Glc0-120_ |
| --- | --- | --- | --- | --- | --- | --- | --- | --- |
| *ARNTL2* | rs7301841 | 0.1 | 0.2 | 0.2 | 0.6 | 0.6 | 0.9 | 0.9 |
| *ARNTL2* | rs10842905 | 0.5 | 0.3 | 0.3 | 0.1 | 0.4 | 0.7 | 0.8 |
| *ARNTL2* | rs7137588 | 0.3 | 0.7 | 0.8 | 0.6 | 0.7 | 0.3 | 0.5 |
| *ARNTL2* | rs11610949 | 0.9 | 0.6 | 0.2 | 0.7 | **0.0410** | 0.7 | 0.8 |
| *ARNTL2* | rs4964052 | 0.9 | 0.3 | 0.6 | 0.09 | 0.1 | 1.0 | 0.7 |
| *ARNTL2* | rs17497683 | 0.9 | 0.8 | 0.4 | 0.9 | 0.09 | 0.3 | 0.4 |
| *ARNTL2* | rs11048977 | 0.8 | 0.9 | 0.2 | 0.3 | 0.5 | 1.0 | 0.9 |
| *ARNTL2* | rs11048978 | 0.9 | 0.6 | 0.9 | 0.2 | 0.3 | 0.6 | 0.7 |
| *ARNTL2* | rs2968756 | 0.4 | 0.3 | 0.8 | 0.4 | 0.08 | 0.7 | 0.7 |
| *ARNTL2* | rs4964055 | 0.5 | 0.8 | 0.2 | 0.9 | 0.1 | 0.8 | 0.7 |
| *ARNTL2* | rs12231701 | 0.09 | 0.1 | 0.06 | 0.4 | 1.0 | 0.9 | 0.7 |
| *ARNTL2* | rs7306410 | **0.0342** | 0.09 | 0.4 | 0.3 | 0.2 | 0.9 | 0.7 |
| *ARNTL2* | rs4964059 | 0.1 | 0.4 | 0.9 | 0.5 | 1.0 | 0.7 | 0.9 |
| *ARNTL2* | rs11048994 | 0.8 | 0.4 | 0.9 | 0.2 | 0.3 | 0.6 | 0.5 |
| *ARNTL2* | rs11048995 | 0.3 | 0.4 | 0.7 | 0.2 | 0.5 | 0.2 | 0.3 |
| *ARNTL2* | rs7304939 | 0.5 | 0.8 | 0.5 | 0.8 | 0.7 | 0.8 | 0.8 |
| *ARNTL2* | rs11048997 | 1.0 | 0.2 | 0.4 | 0.5 | 0.8 | 0.2 | 0.9 |
| *ARNTL2* | rs12319133 | 0.6 | 0.6 | 0.8 | 1.0 | 0.8 | 0.2 | 0.5 |
| *ARNTL2* | rs4409932 | 0.4 | 1.0 | 1.0 | 0.7 | 0.7 | 0.7 | 0.5 |
| *ARNTL2* | rs2306074 | 0.5 | 0.8 | 0.3 | **0.0300** | 0.6 | 0.08 | **0.0329** |
| *ARNTL2* | rs4931075 | 0.3 | 0.1 | 0.6 | **0.0017** | 0.6 | **0.0413** | 0.08 |
| *ARNTL2* | rs11049004 | 0.6 | **0.0353** | 0.4 | 0.2 | 0.9 | 0.5 | 0.4 |
| *ARNTL2* | rs2682706 | 0.2 | 0.1 | 0.6 | **0.0013** | 0.7 | **0.0173** | 0.06 |
| *CLOCK* | rs10462028 | 0.6 | 0.5 | 0.4 | 0.7 | 0.5 | 0.1 | 0.7 |

(continued on next page)

| Gene | SNP | BMI | Body fat content | Glucose (fasting) | 2-h Glucose | ISI (OGTT) | AUC_Ins0-30_/ AUC_Glc0-30_ | AUC_C-Pep0-120_/ AUC_Glc0-120_ |
| --- | --- | --- | --- | --- | --- | --- | --- | --- |
| *CLOCK* | rs1801260 | 0.3 | 0.3 | 0.6 | 0.3 | 0.6 | 0.3 | 0.8 |
| *CLOCK* | rs3792603 | 0.6 | 0.6 | 0.4 | 0.2 | 0.8 | 0.8 | 0.5 |
| *CLOCK* | rs17777927 | 0.3 | 0.1 | 0.1 | 0.5 | 0.8 | 0.1 | 1.0 |
| *CLOCK* | rs4864996 | **0.0459** | 0.4 | 0.09 | 0.4 | 0.7 | 0.3 | 0.9 |
| *CLOCK* | rs11725422 | 0.3 | 0.5 | 0.7 | 0.5 | 1.0 | 0.4 | 0.3 |
| *CLOCK* | rs1554483 | **0.0312** | 0.3 | 0.1 | 0.3 | 0.9 | 0.4 | 1.0 |
| *CLOCK* | rs11932595 | 0.2 | 0.06 | 0.6 | 0.1 | 0.9 | 0.9 | 0.6 |
| *CLOCK* | rs1522113 | 0.4 | 0.5 | 0.7 | 0.4 | 1.0 | 0.3 | 0.3 |
| *CLOCK* | rs11733959 | 0.3 | 0.5 | 0.7 | 0.4 | 1.0 | 0.4 | 0.3 |
| *CLOCK* | rs6554281 | 0.3 | 0.7 | 0.6 | 0.7 | 0.6 | 0.2 | 0.5 |
| *CLOCK* | rs4864548 | **0.0115** | 0.3 | 0.1 | 0.3 | 0.9 | 0.6 | 0.7 |
| *CLOCK* | rs1979604 | 0.3 | 0.3 | 0.7 | 0.4 | 0.6 | 0.2 | 0.9 |
| *CLOCK* | rs726967 | 0.1 | 0.9 | **0.0346** | 0.8 | 0.4 | 0.6 | 1.0 |
| *CRY1* | rs10861688 | 0.9 | 0.3 | 0.5 | 0.09 | 0.2 | 0.4 | 0.8 |
| *CRY1* | rs12368868 | 0.9 | 0.3 | 0.3 | **0.0246** | 0.6 | 0.2 | 0.2 |
| *CRY1* | rs1921126 | 0.4 | 0.9 | 0.6 | 0.6 | 0.5 | 0.2 | 0.4 |
| *CRY1* | rs11113179 | 0.8 | 0.3 | 0.5 | **0.0485** | 0.9 | 0.1 | 0.2 |
| *CRY1* | rs11113181 | 0.9 | 0.4 | 0.6 | **0.0458** | 0.3 | 0.7 | 0.6 |
| *CRY1* | rs17289712 | 0.9 | 0.7 | 0.6 | 0.3 | **0.0386** | 0.5 | 0.4 |
| *CRY2* | rs10838524 | 0.3 | 0.09 | 0.6 | 0.6 | 0.5 | 0.8 | 0.6 |
| *CRY2* | rs11605924 | 0.2 | 0.08 | 0.9 | 0.9 | 0.9 | 0.5 | 0.2 |
| *CRY2* | rs1401417 | 0.3 | 0.07 | 0.9 | 0.3 | 0.5 | 0.5 | 0.8 |
| *CRY2* | rs7123390 | 0.2 | 0.09 | 0.4 | 0.1 | 0.3 | 0.2 | 0.3 |
| *CRY2* | rs7933420 | 0.07 | **0.0316** | 1.0 | 0.6 | 0.9 | 0.5 | 0.1 |

(continued on next page)

| Gene | SNP | BMI | Body fat content | Glucose (fasting) | 2-h Glucose | ISI (OGTT) | AUC_Ins0-30_/ AUC_Glc0-30_ | AUC_C-Pep0-120_/ AUC_Glc0-120_ |
| --- | --- | --- | --- | --- | --- | --- | --- | --- |
| *CRY2* | rs10838527 | 0.7 | 0.4 | 1.0 | 0.7 | 0.7 | 0.6 | 0.06 |
| *CRY2* | rs2292910 | 0.05 | **0.0304** | 0.5 | 0.4 | 0.7 | 0.3 | 0.5 |
| *CRY2* | rs6798 | 0.2 | 0.1 | 0.4 | 0.2 | 0.7 | 0.3 | 0.2 |
| *CRY2* | rs3824872 | 0.8 | 0.7 | 0.2 | 0.06 | 0.2 | 0.5 | 0.7 |
| *CRY2* | rs1554338 | 1.0 | 0.6 | 0.5 | 0.5 | 0.8 | 0.4 | 0.07 |
| *PER1* | rs9914077 | 0.8 | 0.7 | 0.4 | 0.5 | 0.7 | 0.9 | 0.5 |
| *PER1* | rs2289591 | 0.4 | 0.2 | 1.0 | 0.9 | 0.3 | 0.6 | 0.9 |
| *PER1* | rs2735611 | 0.6 | 0.5 | 0.4 | 1.0 | 0.8 | 0.4 | 0.8 |
| *PER1* | rs3027188 | 0.8 | 0.9 | 0.1 | 0.7 | 0.4 | 0.6 | 0.7 |
| *PER1* | rs2304911 | 0.3 | 0.6 | 0.3 | 0.9 | 0.7 | 0.2 | 0.5 |
| *PER1* | rs2518023 | 0.9 | 0.2 | 0.6 | 0.9 | 0.2 | **0.0260** | 0.9 |
| *PER2* | rs881933 | 0.8 | 0.7 | 1.0 | 0.7 | 0.1 | 0.2 | 0.2 |
| *PER2* | rs934945 | 0.3 | 0.1 | 0.8 | 0.7 | 0.1 | 0.7 | 0.9 |
| *PER2* | rs2304670 | 0.2 | 0.1 | 0.8 | 0.8 | 0.7 | 0.7 | 0.9 |
| *PER2* | rs2304669 | 0.5 | 0.1 | 0.8 | 0.9 | 0.9 | 0.1 | 0.4 |
| *PER2* | rs7570188 | **0.0453** | **0.0142** | 0.8 | 0.7 | 1.0 | 0.4 | 0.3 |
| *PER2* | rs3739064 | 0.2 | **0.0492** | 0.6 | 0.5 | 0.9 | 0.1 | 0.2 |
| *PER2* | rs11894535 | 0.4 | 0.8 | 0.7 | 0.4 | 1.0 | 0.1 | 0.2 |
| *PER2* | rs10462023 | 0.8 | 0.9 | 0.6 | 0.8 | 0.2 | 0.8 | 0.5 |
| *PER2* | rs2304673 | 0.7 | 0.3 | 0.3 | 0.8 | 0.8 | **0.0419** | 0.2 |
| *PER2* | rs11892306 | 0.9 | 0.5 | 0.5 | 0.2 | 0.7 | 0.1 | 0.1 |
| *PER2* | rs11894491 | 0.4 | 0.3 | 0.9 | 0.2 | 0.06 | 0.2 | 0.5 |
| *PER3* | rs875994 | 0.5 | 0.4 | 1.0 | 0.1 | 0.7 | 0.7 | 0.5 |
| *PER3* | rs228682 | 0.6 | 1.0 | 0.7 | 0.8 | 0.5 | 0.3 | 0.3 |

(continued on next page)

| Gene | SNP | BMI | Body fat content | Glucose (fasting) | 2-h Glucose | ISI (OGTT) | AUC_Ins0-30_/ AUC_Glc0-30_ | AUC_C-Pep0-120_/ AUC_Glc0-120_ |
| --- | --- | --- | --- | --- | --- | --- | --- | --- |
| *PER3* | rs228666 | 0.4 | 0.2 | 0.8 | 0.7 | 0.8 | 0.8 | 0.4 |
| *PER3* | rs1891217 | 0.9 | 0.5 | 0.8 | 0.05 | 0.1 | 0.3 | 0.07 |
| *PER3* | rs2172563 | 0.8 | 0.5 | 0.7 | 0.2 | 1.0 | 0.6 | 0.6 |
| *PER3* | rs12061787 | 0.2 | 0.6 | 0.3 | 0.3 | 0.5 | 0.5 | 0.2 |
| *PER3* | rs2640908 | 0.3 | 0.3 | 0.5 | 0.8 | 0.5 | 0.8 | 0.9 |
| *PER3* | rs228675 | 0.4 | 0.2 | 0.9 | 0.8 | 0.8 | 0.8 | 0.4 |
| *TIMELESS* | rs17441402 | 0.5 | 0.2 | 0.4 | 1.0 | 0.3 | 0.3 | 0.06 |
| *TIMELESS* | rs4759206 | 0.9 | 0.7 | 1.0 | 0.8 | 0.5 | 0.4 | 0.4 |
| *TIMELESS* | rs2291738 | 0.4 | 1.0 | 0.1 | 0.6 | 0.6 | 0.1 | 1.0 |
| *TIMELESS* | rs774049 | 0.3 | 0.7 | 0.5 | 0.1 | 0.1 | 0.3 | 0.1 |
| *TIMELESS* | rs774035 | 0.6 | 0.9 | 0.3 | 0.5 | 1.0 | 0.4 | 0.5 |
| *TIMELESS* | rs11171846 | 0.8 | 0.8 | 0.5 | 0.1 | 0.3 | 0.2 | 0.7 |
| *TIMELESS* | rs11171852 | 0.7 | 0.9 | 0.4 | 0.5 | 0.6 | 0.5 | 0.9 |
| *TIMELESS* | rs4630333 | 0.8 | 0.7 | 0.1 | 0.9 | 0.6 | 0.2 | 0.9 |
| *TIMELESS* | rs774044 | 0.3 | 0.4 | 0.3 | **0.0423** | **0.0351** | 0.2 | 0.5 |

Data represent p-values for interaction (cross) effects between SNP genotype (additive inheritance model) and gender on the indicated metabolic traits using multiple linear regression analyses (standard least squares method) with (i) age as covariate when assessing interaction effects on BMI/body fat content, (ii) age and BMI as covariates when assessing interaction effects on glycemia/insulin sensitivity, and (iii) age, BMI, and insulin sensitivity as covariates when assessing interaction effects on insulin secretion. Nominal associations (p<0.05) are marked by using bold fonts. AUC – area under the curve; BMI – body mass index; C-Pep – C-peptide; Glc – glucose; Ins – insulin; ISI – insulin sensitivity index; OGTT – oral glucose tolerance test; SNP – single nucleotide polymorphism
