# Supplementary material for: Glucose-Raising Polymorphisms in the Human Clock Gene Cryptochrome 2 (CRY2) Affect Hepatic Lipid Content
Source: PLoS One. 2016 Jan 4;11(1):e0145563. doi: 10.1371/journal.pone.0145563 (PMC4699770; doi:10.1371/journal.pone.0145563)
Supplement: S6 Table — (DOC) [file pone.0145563.s006.doc]

**Table S6. SNP-BMI interaction effects on glucose concentrations, insulin sensitivity, and insulin secretion**

| Gene | SNP | Glucose (fasting) | 2-h Glucose | ISI (OGTT) | AUC_Ins0-30_/ AUC_Glc0-30_ | AUC_C-Pep0-120_/ AUC_Glc0-120_ |
| --- | --- | --- | --- | --- | --- | --- |
| *ARNTL* | rs7112233 | 0.8 | 0.9 | 0.8 | 0.5 | 0.7 |
| *ARNTL* | rs7117492 | 0.9 | 0.6 | 0.5 | 0.8 | 0.9 |
| *ARNTL* | rs12795287 | 0.9 | 0.7 | 0.9 | 0.6 | 0.8 |
| *ARNTL* | rs11022724 | 0.9 | 0.9 | 0.6 | 0.8 | 0.6 |
| *ARNTL* | rs2279284 | 0.2 | 0.8 | 0.9 | 0.6 | 0.6 |
| *ARNTL* | rs7950226 | 0.6 | 0.3 | 0.6 | 0.5 | 0.1 |
| *ARNTL* | rs10766074 | **0.0172** | 0.9 | 0.7 | 0.2 | 0.2 |
| *ARNTL* | rs4757143 | 0.9 | 0.9 | 0.8 | 0.7 | 0.2 |
| *ARNTL* | rs4757144 | 0.8 | 0.7 | 0.8 | 0.8 | 0.5 |
| *ARNTL* | rs6486122 | 0.3 | 0.06 | 0.9 | 1.0 | 0.3 |
| *ARNTL* | rs7937060 | 0.3 | 0.08 | 0.8 | 0.7 | 0.1 |
| *ARNTL* | rs1562438 | **0.0405** | **0.0040** | 0.6 | 0.2 | 0.06 |
| *ARNTL* | rs2290036 | 0.7 | 1.0 | 0.6 | 0.3 | 0.7 |
| *ARNTL* | rs2290037 | 0.5 | 0.5 | 0.3 | 0.5 | 0.4 |
| *ARNTL* | rs1868049 | 0.3 | 0.1 | 0.9 | 0.2 | 0.08 |
| *ARNTL* | rs11022778 | 0.7 | 0.4 | 0.9 | 0.3 | 0.3 |
| *ARNTL* | rs3816358 | 0.9 | 0.1 | 0.7 | 0.4 | 0.4 |
| *ARNTL* | rs4757151 | 0.5 | 0.6 | 0.5 | 0.1 | 0.2 |
| *ARNTL* | rs11600996 | 0.06 | 0.1 | **0.0283** | **0.0427** | 0.1 |
| *ARNTL* | rs10766079 | 0.2 | 0.2 | 0.9 | **0.0036** | 0.1 |
| *ARNTL* | rs969485 | 0.3 | 0.4 | 0.5 | 0.06 | 0.5 |
| *ARNTL* | rs11022783 | 0.3 | 0.3 | 1.0 | 0.05 | 0.3 |
| *ARNTL* | rs10832031 | 0.3 | 0.5 | 0.3 | 0.7 | 0.3 |

(continued on next page)

| Gene | SNP | Glucose (fasting) | 2-h Glucose | ISI (OGTT) | AUC_Ins0-30_/ AUC_Glc0-30_ | AUC_C-Pep0-120_/ AUC_Glc0-120_ |
| --- | --- | --- | --- | --- | --- | --- |
| *ARNTL2* | rs7301841 | 0.9 | 0.5 | 0.8 | 0.4 | 0.6 |
| *ARNTL2* | rs10842905 | 0.1 | **0.0476** | 0.5 | **0.0241** | 0.1 |
| *ARNTL2* | rs7137588 | 0.8 | 0.2 | **0.0290** | 0.4 | 0.7 |
| *ARNTL2* | rs11610949 | 0.2 | 0.8 | 0.3 | **0.0320** | 0.1 |
| *ARNTL2* | rs4964052 | 0.9 | 1.0 | 0.7 | 0.9 | 0.9 |
| *ARNTL2* | rs17497683 | 0.3 | **0.0166** | 0.1 | 0.09 | 0.1 |
| *ARNTL2* | rs11048977 | 0.4 | **0.0150** | 0.6 | 0.2 | 0.1 |
| *ARNTL2* | rs11048978 | 1.0 | 0.8 | 0.3 | 0.7 | 0.9 |
| *ARNTL2* | rs2968756 | 0.2 | **0.0120** | **0.0436** | 0.6 | 0.7 |
| *ARNTL2* | rs4964055 | 0.1 | 0.7 | 0.2 | 0.8 | 0.8 |
| *ARNTL2* | rs12231701 | 0.9 | 0.6 | 0.7 | 0.4 | 0.7 |
| *ARNTL2* | rs7306410 | 0.8 | 0.07 | 0.8 | 0.2 | 0.3 |
| *ARNTL2* | rs4964059 | 0.5 | 0.9 | 0.1 | 0.8 | 0.9 |
| *ARNTL2* | rs11048994 | 1.0 | 0.8 | 0.6 | 0.7 | 0.6 |
| *ARNTL2* | rs11048995 | 0.8 | 0.05 | 0.2 | 1.0 | 0.08 |
| *ARNTL2* | rs7304939 | 0.9 | 0.2 | 0.7 | 0.4 | 0.8 |
| *ARNTL2* | rs11048997 | 0.7 | 0.06 | 0.3 | 0.4 | 0.2 |
| *ARNTL2* | rs12319133 | 1.0 | 0.1 | 1.0 | 0.2 | 0.2 |
| *ARNTL2* | rs4409932 | 0.7 | 0.2 | 0.7 | 1.0 | 0.8 |
| *ARNTL2* | rs2306074 | 0.8 | 0.4 | 0.8 | 1.0 | 0.7 |
| *ARNTL2* | rs4931075 | 0.1 | 0.7 | 0.3 | 0.5 | 0.5 |
| *ARNTL2* | rs11049004 | **0.0465** | 0.2 | 0.5 | 0.05 | 0.1 |
| *ARNTL2* | rs2682706 | 0.1 | 0.9 | 0.3 | 0.2 | 0.2 |
| *CLOCK* | rs10462028 | 0.5 | 0.8 | 0.3 | 0.7 | 0.5 |

(continued on next page)

| Gene | SNP | Glucose (fasting) | 2-h Glucose | ISI (OGTT) | AUC_Ins0-30_/ AUC_Glc0-30_ | AUC_C-Pep0-120_/ AUC_Glc0-120_ |
| --- | --- | --- | --- | --- | --- | --- |
| *CLOCK* | rs1801260 | 0.5 | 0.9 | 0.4 | 0.8 | 0.5 |
| *CLOCK* | rs3792603 | 0.2 | 0.4 | 0.3 | 0.2 | 0.8 |
| *CLOCK* | rs17777927 | 0.4 | 0.5 | 0.08 | 0.2 | 0.6 |
| *CLOCK* | rs4864996 | 0.9 | 0.1 | 0.4 | 0.3 | 0.6 |
| *CLOCK* | rs11725422 | 1.0 | 0.2 | 0.8 | 1.0 | 0.7 |
| *CLOCK* | rs1554483 | 0.9 | 0.3 | 0.5 | 0.5 | 0.9 |
| *CLOCK* | rs11932595 | 0.3 | 0.2 | 0.05 | 0.4 | 0.8 |
| *CLOCK* | rs1522113 | 1.0 | 0.3 | 0.8 | 0.9 | 0.7 |
| *CLOCK* | rs11733959 | 1.0 | 0.2 | 0.8 | 1.0 | 0.7 |
| *CLOCK* | rs6554281 | 0.5 | 0.6 | 0.5 | 0.3 | 0.5 |
| *CLOCK* | rs4864548 | 0.9 | 0.3 | 0.5 | 0.3 | 0.8 |
| *CLOCK* | rs1979604 | 0.4 | 0.7 | 0.2 | 0.7 | 0.6 |
| *CLOCK* | rs726967 | 0.4 | 0.1 | **0.0466** | 0.2 | 0.7 |
| *CRY1* | rs10861688 | 0.9 | 0.6 | 0.09 | 0.5 | 1.0 |
| *CRY1* | rs12368868 | 0.7 | 0.08 | 0.7 | 0.5 | 0.2 |
| *CRY1* | rs1921126 | 0.9 | 0.4 | 1.0 | 0.6 | 1.0 |
| *CRY1* | rs11113179 | 0.7 | 0.07 | 0.9 | 0.6 | 0.3 |
| *CRY1* | rs11113181 | 0.8 | 0.4 | 0.09 | 0.6 | 0.8 |
| *CRY1* | rs17289712 | 1.0 | 0.4 | 0.1 | 0.6 | 0.8 |
| *CRY2* | rs10838524 | 0.09 | 0.7 | 0.1 | 0.1 | 0.5 |
| *CRY2* | rs11605924 | 0.1 | 1.0 | 0.1 | 0.1 | 0.4 |
| *CRY2* | rs1401417 | **0.0233** | 0.1 | 0.1 | 0.7 | 1.0 |
| *CRY2* | rs7123390 | **0.0325** | 0.4 | 0.1 | 0.7 | 0.9 |
| *CRY2* | rs7933420 | 0.1 | 1.0 | 0.2 | 0.2 | 0.5 |

(continued on next page)

| Gene | SNP | Glucose (fasting) | 2-h Glucose | ISI (OGTT) | AUC_Ins0-30_/ AUC_Glc0-30_ | AUC_C-Pep0-120_/ AUC_Glc0-120_ |
| --- | --- | --- | --- | --- | --- | --- |
| *CRY2* | rs10838527 | 0.1 | **0.0137** | 0.2 | **0.0344** | 0.2 |
| *CRY2* | rs2292910 | **0.0248** | 0.1 | **0.0183** | 0.9 | 0.9 |
| *CRY2* | rs6798 | 0.6 | 0.8 | 0.4 | 0.8 | 0.6 |
| *CRY2* | rs3824872 | 0.7 | 0.2 | 0.9 | 0.2 | 0.4 |
| *CRY2* | rs1554338 | 0.7 | 0.9 | 0.9 | 0.6 | 1.0 |
| *PER1* | rs9914077 | 0.4 | 0.09 | 0.6 | 0.5 | 0.5 |
| *PER1* | rs2289591 | 0.4 | 0.1 | 0.6 | 0.8 | 0.6 |
| *PER1* | rs2735611 | 1.0 | 1.0 | 0.2 | 0.7 | 0.9 |
| *PER1* | rs3027188 | 0.7 | 0.7 | 0.8 | 0.7 | 0.8 |
| *PER1* | rs2304911 | 0.3 | **0.0295** | 0.2 | **0.0267** | 0.1 |
| *PER1* | rs2518023 | 0.5 | 0.4 | 0.5 | 1.0 | 0.4 |
| *PER2* | rs881933 | 0.5 | 0.3 | 0.7 | 0.8 | 0.7 |
| *PER2* | rs934945 | 0.08 | 0.7 | 0.4 | 0.5 | 0.4 |
| *PER2* | rs2304670 | 0.5 | **0.0411** | 0.1 | 0.7 | 0.5 |
| *PER2* | rs2304669 | 0.4 | 0.07 | 0.8 | 0.9 | 0.3 |
| *PER2* | rs7570188 | 0.7 | 0.3 | 0.07 | 0.5 | 0.8 |
| *PER2* | rs3739064 | 0.3 | 0.7 | 0.5 | 0.4 | 0.7 |
| *PER2* | rs11894535 | 0.2 | **0.0035** | **0.0425** | 0.9 | 0.3 |
| *PER2* | rs10462023 | 0.3 | 0.6 | 0.2 | 0.6 | 0.4 |
| *PER2* | rs2304673 | 0.6 | 0.06 | 1.0 | 0.7 | 0.4 |
| *PER2* | rs11892306 | 0.2 | 0.3 | 0.7 | 0.5 | 0.5 |
| *PER2* | rs11894491 | **0.0325** | 0.5 | 0.08 | 0.2 | 0.2 |
| *PER3* | rs875994 | 0.8 | 0.7 | 0.5 | 0.9 | 0.7 |
| *PER3* | rs228682 | 0.4 | 0.8 | 0.7 | 0.5 | 0.6 |

(continued on next page)

| Gene | SNP | Glucose (fasting) | 2-h Glucose | ISI (OGTT) | AUC_Ins0-30_/ AUC_Glc0-30_ | AUC_C-Pep0-120_/ AUC_Glc0-120_ |
| --- | --- | --- | --- | --- | --- | --- |
| *PER3* | rs228666 | 0.3 | 0.8 | 0.9 | 0.7 | 0.9 |
| *PER3* | rs1891217 | 0.8 | 0.4 | 0.3 | 0.2 | 0.7 |
| *PER3* | rs2172563 | 0.5 | 0.3 | 0.7 | 0.8 | 0.9 |
| *PER3* | rs12061787 | 0.3 | 0.5 | 0.7 | 0.3 | 1.0 |
| *PER3* | rs2640908 | 0.5 | 0.6 | 0.09 | 0.8 | 1.0 |
| *PER3* | rs228675 | 0.3 | 0.9 | 0.8 | 0.8 | 1.0 |
| *TIMELESS* | rs17441402 | **0.0314** | 0.4 | 0.9 | **0.0460** | 0.2 |
| *TIMELESS* | rs4759206 | **0.0190** | 1.0 | **0.0234** | 0.6 | 0.6 |
| *TIMELESS* | rs2291738 | 0.5 | 0.1 | **0.0200** | 0.3 | 0.8 |
| *TIMELESS* | rs774049 | 0.2 | 0.5 | 0.5 | 0.5 | 0.8 |
| *TIMELESS* | rs774035 | 0.5 | 0.1 | 0.08 | 0.5 | 0.8 |
| *TIMELESS* | rs11171846 | 0.6 | 0.2 | 0.4 | 0.08 | 0.8 |
| *TIMELESS* | rs11171852 | 0.1 | 0.3 | 0.1 | 0.09 | 0.5 |
| *TIMELESS* | rs4630333 | 0.4 | 0.07 | **0.0155** | 0.1 | 0.8 |
| *TIMELESS* | rs774044 | 0.2 | 0.1 | 0.1 | 0.9 | 0.6 |

Data represent p-values for interaction (cross) effects between SNP genotype (additive inheritance model) and BMI on the indicated metabolic traits using multiple linear regression analyses (standard least squares method) with (i) gender and age as covariates when assessing interaction effects on glycemia/insulin sensitivity and (ii) gender, age, and insulin sensitivity as covariates when assessing interaction effects on insulin secretion. Nominal associations (p<0.05) are marked by using bold fonts. AUC – area under the curve; BMI – body mass index; C-Pep – C-peptide; Glc – glucose; Ins – insulin; ISI – insulin sensitivity index; OGTT – oral glucose tolerance test; SNP – single nucleotide polymorphism
