# Supplementary material for: Glucose-Raising Polymorphisms in the Human Clock Gene Cryptochrome 2 (CRY2) Affect Hepatic Lipid Content
Source: PLoS One. 2016 Jan 4;11(1):e0145563. doi: 10.1371/journal.pone.0145563 (PMC4699770; doi:10.1371/journal.pone.0145563)
Supplement: S7 Table — (DOC) [file pone.0145563.s007.doc]

**Table S7. SNP-glucose interaction effects on insulin secretion**

| Gene | SNP | AUC_Ins0-30_/ AUC_Glc0-30_ | AUC_C-Pep0-120_/ AUC_Glc0-120_ |
| --- | --- | --- | --- |
| *ARNTL* | rs7112233 | 0.8 | 0.7 |
| *ARNTL* | rs7117492 | **0.0228** | 0.2 |
| *ARNTL* | rs12795287 | 0.3 | 0.6 |
| *ARNTL* | rs11022724 | 0.5 | 0.3 |
| *ARNTL* | rs2279284 | 0.2 | 0.7 |
| *ARNTL* | rs7950226 | **0.0131** | 0.6 |
| *ARNTL* | rs10766074 | 0.2 | 0.7 |
| *ARNTL* | rs4757143 | 0.3 | 0.8 |
| *ARNTL* | rs4757144 | 0.6 | 0.8 |
| *ARNTL* | rs6486122 | 0.2 | 0.7 |
| *ARNTL* | rs7937060 | 0.9 | 0.8 |
| *ARNTL* | rs1562438 | 1.0 | 1.0 |
| *ARNTL* | rs2290036 | 0.3 | 0.9 |
| *ARNTL* | rs2290037 | 0.3 | 0.3 |
| *ARNTL* | rs1868049 | **0.0156** | 0.2 |
| *ARNTL* | rs11022778 | **0.0282** | 0.9 |
| *ARNTL* | rs3816358 | 0.5 | 0.5 |
| *ARNTL* | rs4757151 | 0.06 | 0.2 |
| *ARNTL* | rs11600996 | 1.0 | 0.8 |
| *ARNTL* | rs10766079 | **0.0069** | 0.1 |
| *ARNTL* | rs969485 | 0.6 | 0.5 |
| *ARNTL* | rs11022783 | **0.0114** | **0.0451** |
| *ARNTL* | rs10832031 | 0.3 | 0.4 |

(continued on next page)

| Gene | SNP | AUC_Ins0-30_/ AUC_Glc0-30_ | AUC_C-Pep0-120_/ AUC_Glc0-120_ |
| --- | --- | --- | --- |
| *ARNTL2* | rs7301841 | 0.7 | 0.6 |
| *ARNTL2* | rs10842905 | 0.6 | 0.4 |
| *ARNTL2* | rs7137588 | 0.9 | 1.0 |
| *ARNTL2* | rs11610949 | 0.4 | 0.8 |
| *ARNTL2* | rs4964052 | 0.9 | 0.6 |
| *ARNTL2* | rs17497683 | 0.4 | 0.5 |
| *ARNTL2* | rs11048977 | 0.4 | 0.3 |
| *ARNTL2* | rs11048978 | 0.3 | 0.4 |
| *ARNTL2* | rs2968756 | 0.3 | 0.7 |
| *ARNTL2* | rs4964055 | 0.2 | 0.7 |
| *ARNTL2* | rs12231701 | 0.9 | 0.6 |
| *ARNTL2* | rs7306410 | 0.7 | 0.8 |
| *ARNTL2* | rs4964059 | 0.9 | 0.9 |
| *ARNTL2* | rs11048994 | 0.7 | 0.4 |
| *ARNTL2* | rs11048995 | 0.07 | 0.1 |
| *ARNTL2* | rs7304939 | 0.8 | 0.07 |
| *ARNTL2* | rs11048997 | 0.6 | 0.06 |
| *ARNTL2* | rs12319133 | 0.4 | 0.1 |
| *ARNTL2* | rs4409932 | 0.5 | 0.08 |
| *ARNTL2* | rs2306074 | 0.4 | 0.9 |
| *ARNTL2* | rs4931075 | 0.3 | 0.1 |
| *ARNTL2* | rs11049004 | 0.7 | 0.05 |
| *ARNTL2* | rs2682706 | 0.4 | 0.2 |
| *CLOCK* | rs10462028 | 0.3 | 0.9 |

(continued on next page)

| Gene | SNP | AUC_Ins0-30_/ AUC_Glc0-30_ | AUC_C-Pep0-120_/ AUC_Glc0-120_ |
| --- | --- | --- | --- |
| *CLOCK* | rs1801260 | 1.0 | 0.2 |
| *CLOCK* | rs3792603 | 0.6 | 0.3 |
| *CLOCK* | rs17777927 | 0.6 | 0.9 |
| *CLOCK* | rs4864996 | 0.7 | 0.5 |
| *CLOCK* | rs11725422 | **0.0074** | **0.0211** |
| *CLOCK* | rs1554483 | 0.5 | 0.09 |
| *CLOCK* | rs11932595 | 0.8 | 0.4 |
| *CLOCK* | rs1522113 | **0.0072** | **0.0207** |
| *CLOCK* | rs11733959 | **0.0070** | **0.0198** |
| *CLOCK* | rs6554281 | 0.5 | 0.1 |
| *CLOCK* | rs4864548 | 0.3 | 0.07 |
| *CLOCK* | rs1979604 | 0.8 | 0.2 |
| *CLOCK* | rs726967 | 0.4 | 0.5 |
| *CRY1* | rs10861688 | 0.7 | **0.0062** |
| *CRY1* | rs12368868 | 0.8 | 0.6 |
| *CRY1* | rs1921126 | 0.8 | **0.0493** |
| *CRY1* | rs11113179 | 0.6 | 0.5 |
| *CRY1* | rs11113181 | 0.8 | **0.0008** |
| *CRY1* | rs17289712 | **0.0290** | 0.7 |
| *CRY2* | rs10838524 | 0.3 | 0.7 |
| *CRY2* | rs11605924 | 0.2 | 0.7 |
| *CRY2* | rs1401417 | 0.5 | 0.3 |
| *CRY2* | rs7123390 | 0.4 | 0.2 |
| *CRY2* | rs7933420 | 0.2 | 1.0 |

(continued on next page)

| Gene | SNP | AUC_Ins0-30_/ AUC_Glc0-30_ | AUC_C-Pep0-120_/ AUC_Glc0-120_ |
| --- | --- | --- | --- |
| *CRY2* | rs10838527 | 1.0 | 0.1 |
| *CRY2* | rs2292910 | 0.1 | 0.7 |
| *CRY2* | rs6798 | 0.9 | 0.8 |
| *CRY2* | rs3824872 | 0.7 | 0.2 |
| *CRY2* | rs1554338 | 0.2 | 0.4 |
| *PER1* | rs9914077 | 0.07 | 0.7 |
| *PER1* | rs2289591 | 0.1 | 0.8 |
| *PER1* | rs2735611 | 0.6 | 0.5 |
| *PER1* | rs3027188 | 0.2 | 0.3 |
| *PER1* | rs2304911 | 0.6 | 0.3 |
| *PER1* | rs2518023 | 0.4 | **0.0473** |
| *PER2* | rs881933 | 0.1 | 0.6 |
| *PER2* | rs934945 | 0.2 | 0.3 |
| *PER2* | rs2304670 | **0.0247** | **0.0065** |
| *PER2* | rs2304669 | 0.2 | 0.9 |
| *PER2* | rs7570188 | 0.8 | 1.0 |
| *PER2* | rs3739064 | 0.6 | 0.1 |
| *PER2* | rs11894535 | 0.2 | 1.0 |
| *PER2* | rs10462023 | 0.5 | 0.8 |
| *PER2* | rs2304673 | 0.3 | 0.6 |
| *PER2* | rs11892306 | 0.7 | 0.06 |
| *PER2* | rs11894491 | 0.2 | 0.05 |
| *PER3* | rs875994 | 0.1 | 0.7 |
| *PER3* | rs228682 | 1.0 | 0.5 |

(continued on next page)

| Gene | SNP | AUC_Ins0-30_/ AUC_Glc0-30_ | AUC_C-Pep0-120_/ AUC_Glc0-120_ |
| --- | --- | --- | --- |
| *PER3* | rs228666 | 0.3 | 0.9 |
| *PER3* | rs1891217 | **0.0264** | 0.4 |
| *PER3* | rs2172563 | 0.8 | 0.2 |
| *PER3* | rs12061787 | 0.5 | 0.3 |
| *PER3* | rs2640908 | 0.9 | 0.3 |
| *PER3* | rs228675 | 0.3 | 1.0 |
| *TIMELESS* | rs17441402 | 0.6 | 0.8 |
| *TIMELESS* | rs4759206 | 0.5 | 1.0 |
| *TIMELESS* | rs2291738 | 0.5 | 0.9 |
| *TIMELESS* | rs774049 | 0.8 | 0.7 |
| *TIMELESS* | rs774035 | 0.4 | 0.8 |
| *TIMELESS* | rs11171846 | 0.6 | 0.8 |
| *TIMELESS* | rs11171852 | 0.6 | 0.7 |
| *TIMELESS* | rs4630333 | 0.6 | 0.9 |
| *TIMELESS* | rs774044 | 0.9 | 0.4 |

Data represent p-values for interaction (cross) effects between SNP genotype (additive inheritance model) and AUC glucose on insulin secretion using multiple linear regression analyses (standard least squares method) with gender, age, BMI, and insulin sensitivity as covariates. Nominal associations (p<0.05) are marked by using bold fonts. AUC – area under the curve; BMI – body mass index; C-Pep – C-peptide; Glc – glucose; Ins – insulin; SNP – single nucleotide polymorphism
